# Characterization of *Sideritis clandestina* subsp. *peloponnesiaca* Polar Glycosides and Phytochemical Comparison to Other Mountain Tea Populations

**DOI:** 10.3390/molecules27217613

**Published:** 2022-11-06

**Authors:** Virginia D. Dimaki, Konstantina Zeliou, Fotini Nakka, Michaela Stavreli, Ioannis Bakratsas, Ligeri Papaioannou, Gregoris Iatrou, Fotini N. Lamari

**Affiliations:** 1 Laboratory of Pharmacognosy & Chemistry of Natural Products, Department of Pharmacy, University of Patras, 26504 Patras, Greece; 2 Division of Plant Biology, Department of Biology, University of Patras, 26504 Patras, Greece

**Keywords:** mountain tea, Lamiaceae, iridoids, phenylethanoids, metabolomics, ultrasound-assisted extraction, flavonoids, melittoside, ajugoside, verbascoside

## Abstract

*Sideritis clandestina* (Bory & Chaub.) Hayek subsp. *peloponnesiaca* (Boiss. & Heldr.) Baden (SCP) is endemic to the mountains of the Northern Peloponnese (Greece). This and other *Sideritis* taxa, collectively known as mountain tea, are widely ingested as beverages for refreshment or medicinal purposes. We describe a methodology for the characterization of SCP. Four iridoid glycosides (monomelittoside, melittoside, ajugoside, and 7-O-acetyl-8-epiloganic acid), two phenolic acid glycosides (vanillic and salicylic acid glycosides), and three caffeoyl ester glycosides (chlorogenic acid, verbascoside, and isoverbascoside) were isolated from SCP for the first time. We used ultrasound-assisted extraction of 3 g of plant material to produce petroleum ether and aqueous extracts, which we then analyzed using GC/MS and LC/MS. This was applied to eight samples from four different taxa. In total, 70 volatile and 27 polar metabolites were determined. The *S. clandestina* samples had a lower phenolic content and weaker antioxidant properties than *S. raeseri* and *S. scardica*. However, *S. clandestina* ssp. *clandestina* seemed to be the most aromatic taxon, with almost double the number of volatiles as the others. Τhis study could contribute to authentication and chemotaxonomic studies of *Sideritis* taxa.

## 1. Introduction

The genus *Sideritis* (Lamiaceae family) comprises more than 150 species worldwide [1]. They are annual or perennial xerophytic and thermophytic shrubs growing in mountain areas. Most of the *Sideritis* species are consumed as infusions of exquisite aroma and taste and are widely known as mountain tea. In traditional medicine, infusions and decoctions of its aerial parts are used as a remedy for cough, common cold, pain, asthma, gastrointestinal disorders, and mild anxiety. Meanwhile, the increasing number of studies on their bioactivity is confirming those actions and is revealing numerous bioactive phytoconstituents [2,3]. The commercial demand for large quantities of mountain tea as a raw material for the production of beverages, cosmetics, herbal drugs, and food supplements, and the research findings on its beneficial health properties, intensify the efforts of cultivation, plant breeding, and authentication of the plant material.

Their taxonomical classification is difficult due to their tendency to hybridize, and many chemotaxonomic approaches have been adopted [1,4,5,6,7]. Apart from the chemovariability that derives not only from the genotype but also from the location, the time of collection, the environmental conditions, and cultivation practices, there is an intrinsic difficulty in those phytochemical studies since the genus *Sideritis* is a rich source of secondary metabolites [1]. For most of the *Sideritis* taxa, their phytochemistry is largely unknown and new natural products are discovered annually.

Most studies have focused on essential oil chemistry, but *Sideritis* taxa have a relatively low content of essential oil; the monoterpenes α-pinene and β-pinene are usually present in high concentrations in the majority of *Sideritis* taxa [1]. Regarding the non-volatiles, quinic acid derivatives, flavonoids and their glucosides, phenylpropanoid glucosides such as verbascoside and isoverbascoside, and some iridoids, with melittoside being the most usual one, were present in all studied *Sideritis* plants [4,6,8].

*Sideritis clandestina* (Bory & Chaub.) Hayek is a variable hemicryptophytic species endemic on the mountains of the Peloponnese, prospering at altitudes of 1600–2300 m. Taxonomically, two subspecies can be recognized: *S. clandestina* (Bory & Chaub.) Hayek subsp. *peloponnesiaca* (Boiss. & Heldr.) Baden (SCP), endemic to the mountains of Central and Northern Peloponnese, and *S. clandestina* (Bory & Chaub.) Hayek subsp. *clandestina* (SCC), which prospers in the southern mountains, on Taygetos and Parnon [9] (pp. 84–91). The variability of the species is expressed by synonyms that have been given in previous times to several populations of the species.

Synonyms of SCP are *Sideritis peloponnesiaca* Boiss. & Heldr., *Sideritis theezans* subsp. *peloponnesiaca* (Boiss. & Heldr.) Bornm., and *Sideritis clandestina* subsp. *cyllenea* (Boiss.) Papan. & Kokkini, whereas synonyms of SCC are *Phlomis clandestina* Bory & Chaub., *Sideritis cretica* Sm., *Sideritis syriaca* Bory & Chaub., and *Sideritis theezans* Boiss. & Heldr. [9] (pp. 84–91). There are studies on their essential-oil composition [10,11,12,13]. We have previously demonstrated that SCP and SCC tea consumption enhances the antioxidant defense of the adult rodent brain in a region-specific manner [14,15] and that SCC confers anxiolysis to rodents [15]. The LC/MS characterization of SCC infusion showed the presence of 17 compounds, including quinic acid and melittoside derivatives, martynoside and β-hydroxyverbascoside, and apigenin and isoscutellarein glycosides [15]. However, several peaks in the SCC extract could not be characterized, and there has not been any analysis of SCP. In 2017, we evaluated several distillation and extraction methods and reached the conclusion that petroleum ether ultrasound-assisted extraction of a small amount of plant material after acidic pretreatment could facilitate a thorough determination of volatiles in SCP and other *Sideritis* taxa [16].

With the aim of characterizing the phytochemical profile of SCP for the first time, we proceeded to the fractionation and isolation of pure polar glycosides from SCP and developed a methodology of metabolomic fingerprinting to compare with similar taxa and contribute to the authentication and chemotaxonomic efforts. In particular, in order to record both volatiles and polar metabolites from a small amount of plant material, we revisited the extraction method developed earlier [16] and analyzed both aqueous and petroleum ether extracts. We applied this methodology to SCP, SCC, and two other *Sideritis* taxa, i.e., *Sideritis raeseri* Boiss. & Heldr. and *Sideritis scardica* Griseb. *S. raeseri* is a variable species occurring in the southern and western part of the Balkan Peninsula. In Greece, two subspecies can be recognized: subsp. *raeseri* (SR) in Northern Greece up to Sterea Hellas (recently collected from the mountain Gaidourorahi in the Northern Peloponnese) and subsp. *attica* (Heldr.) Papanic. & Kokkini in a few mountains of Sterea Hellas (Pateras, Kitheron, Parnis), whereas *S. scardica* (SS) prospers in the Southern Balkan Peninsula, including Northern Greece, and is cultivated in Southern Greece. In order to compare those taxa, we also applied colorimetric assays to evaluate the antioxidant properties of the aqueous extracts.

## 2. Results and Discussion

### 2.1. Isolation of Polar Glycosides from SCP

**Figure 1:**
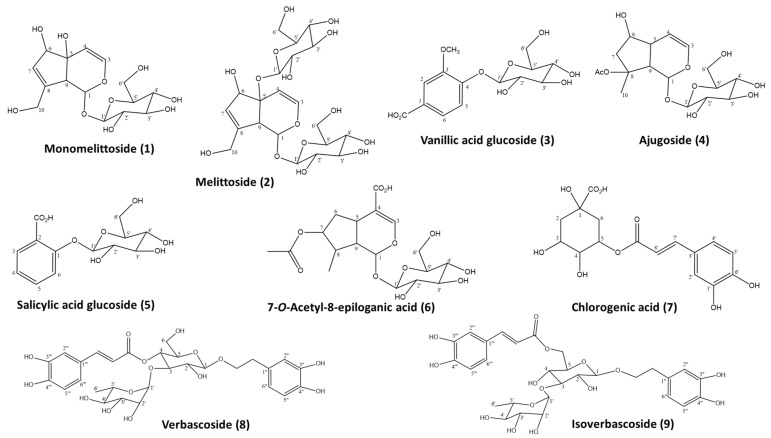
Structures of isolated iridoid, phenylpropanoid, and phenolic acid glycosides.

To facilitate the characterization of SCP metabolites and the qualitative analysis of the aqueous extracts, we proceeded with the isolation of polar compounds from SCP methanolic extract, and the isolated compounds were used as reference compounds. To the best of our knowledge, it is the first time the isolation of nine polar metabolites from SCP species is reported (Figure 1). The molecular structures of the isolated compounds were determined mainly with ^1^H-NMR spectra in comparison with literature data, as well as UV and MS spectra. The small quantities and the poor solubility of the compounds did not give us the proper results from ^13^C -NMR and 2D experiments in all cases.

Monomelittoside (**1**) with the molecular structure C_15_H_22_O_10_ and a molecular weight (M.W.) of 362 was isolated as a pale white solid. The ^1^H-NMR, MS, and UV-vis spectra are presented in Table S1 and Figures S1–S3, and are in accordance with previous studies [17]. It is the first time this iridoid glycoside was isolated from SCP; it has previously been isolated from *S. perfoliata* subsp. *perfoliata* and *S. sipylea* [18,19].

Μelittoside (**2**) was isolated as a white amorphous powder and its molecular structure C_21_H_32_O_15_ was determined by ^1^H- & ^13^C-NMR, MS, and UV-vis spectra (Table S2 and Figures S4–S7), which agreed with a previous reference [17]. This is an iridoid glycoside that bears two glucose units at C-1 and C-5 linked via O-glycosidic bonds and its presence in several *Sideritis* taxa has been reported earlier [1,15,20,21,22]; this is the first report of its occurrence in SCP.

Vanillic acid glucoside (**3**), C_14_H_18_O_9_ and M.W. of 330, was isolated as light grey powder and the structure was determined with ^1^H-NMR, MS, and UV spectra (Table S3 and Figures S8–S10) in accordance with Yu et al. [23]. Vanillic acid presence in *Sideritis* taxa has been reported earlier [24,25] but not of its glucoside.

Ajugoside (**4**), C_17_H_26_O_10_ and M.W. of 390, was isolated as a pale white powder, and the ^1^H-NMR, MS, and UV spectra (Table S4, Figures S11–S13) were in accordance with published data [26]. This iridoid has been isolated from several *Sideritis* taxa like *S. perfoliata* subsp. *perfoliata* [1,27].

Compound (**5**) was isolated as pale viscous solid and it was ascribed to salicylic acid glucoside (**5**), C_13_H_16_O_8_, according to ^1^H-NMR, MS, UV spectra (Table S5 and Figures S14–S16), and literature data [28,29].

A 7-O-acetyl-8-*epi*-loganic acid (**6**) C_18_H_25_O_11_ (M.W. 418) was isolated as a brown sticky solid, and the structure was determined by ^1^H-NMR, MS, and UV-vis spectra (Table S6 and Figures S17–S19), which was in accordance with Hanoglu et al. [30].

Chlorogenic acid (**7**) C_16_H_18_O_9_ (M.W. 354) was isolated as a white amorphous powder and the structure was elucidated from spectroscopic data (^1^H-NMR, MS, and UV-vis spectra in Table S7 and Figures S20–S22) that agreed with the literature [31]. It has been previously isolated from several Mediterranean *Sideritis* taxa [1].

The phenylethanoid glycoside isomers verbascoside (**8**) and isoverbascoside (**9**) C_29_H_36_O_15_ were isolated as an amorphous powder and elucidated using 1D NMR (^1^H, ^13^C, and APT), 2D NMR (HSQC, HMBC, COSY, and ROSEY), and MS spectra in accordance with previous studies [32,33]. The spectroscopic data for verbascoside (**8**) are presented in Table S8 and Figures S23–S30 and those for isoverbascoside (**9**) are presented in Table S9 and Figures S31–S38. References to their occurrence in other *Sideritis* taxa are provided in the comprehensive review by Fraga in 2012 [1].

### 2.2. Qualitative and Quantitative Analysis of Polar Compounds in the Aqueous Extracts by LC/MS Analysis

The acidic pretreatment combined with ultrasound-assisted extraction was previously developed by us and applied to the analysis of volatiles in the petroleum ether extracts [16], but at that time we did not use the aqueous extracts. In this work, we extend the applications of the UAE extraction of a small quantity of plant material (3 g) and describe the characterization of the aqueous extracts, as well. Two biological samples of SCP, SCC, SR, and SS were extracted, and the final yields of the acidic extraction were 30.11%, 35.00%, 39.14%, and 30.29%, respectively. The LC-ESI-MS analysis of the extracts (see Figure S39) combined with the use of the isolated compounds **1**–**9** and rutin as standards, allowed the identification and quantification of most of the components, 27 in total, classifying them into several phytochemical groups: iridoid, quinic acid, phenylpropanoid, and glycosylated flavonoid derivatives (Table 1). Among these groups, flavonoids were the most prevalent one. The results are congruent with many previous studies [6,8,22,34,35,36,37]. The quantitation was performed with a common glycosylated flavonoid, rutin, as an external standard and not with the identified/isolated compounds; the non-commercially available compounds were not isolated in satisfactory amounts, and we did not isolate any flavonoids. The results are expressed as mg rutin equivalents/100 g dry plant material. Despite the anticipated lack of accuracy stemming from the use of a standard that is not present in *Sideritis*, this methodological approach offers the advantage of broad applicability in all laboratories, since rutin is cheap and commercially available.

The highest concentration of polar metabolites was found in the SR extract, followed by SS, whereas SCP had the lowest. Flavonoids were abundant in all four taxa (53.49–170.82 mg/100 g dry plant material in the order SS > SR > SCC and SCP), followed by phenylpropanoids (50.98–164.70 mg/100 g in the order SR > SS > SCC > SCP). Hypolaetin glycosylated derivatives (compounds C14, C19, C20, C21, C23, C24, and C25) had the strongest presence, especially in SR and SS samples (108.02 and 96.73 mg/100 g of dry plant material, respectively). Herein, the distinction between isoscutellarein and luteolin glycosylated derivatives was not feasible in all cases.

Among the four taxa, the most abundant constituent was chlorogenic acid and was followed by the phenylpropanoid verbascoside, which was detected in all samples but quantified in three of them (SCC, SR, and SS samples). Chlorogenic acid, verbascoside, and isoverbascoside presence in SR and SS was reported earlier [8,34,38].

It is noteworthy to comment on the presence of the iridoids melittoside, acetyl-8-epi- loganic acid, and ajugoside. To the best of our knowledge, it is the first time that melittoside, acetyl-8-epi-loganic acid and ajugoside have been determined in SCP and SR taxa; acetyl-8-epi-loganic acid has been identified and isolated only from *Sideritis cypria* [21,30] and ajugoside from *Sideritis perfoliata*, *Sideritis romana*, and *Sideritis cypria* [21,27,39]. Melittoside was previously reported by Vasilopoulou et al. [15] in an SCC aqueous extract but it was not isolated, and was isolated from SS by Koleva et al. [20]. Finally, four unknown compounds were quantified in relatively high concentrations, mainly in SCC extract. Altogether, the highest concentration of iridoids was determined in SR followed by SCP and SCC (43.11, 26.47, and 15.52 mg/100 g of dry plant material, respectively), whereas they were not detected in SS.

### 2.3. Determination of Total Phenolics, Flavonoid Content, and Antioxidant Capacity (DPPH and FRAP) in Aqueous Extracts

The total phenolic content (TPC), total flavonoid content (TFC), and antioxidant capacity (FRAP and DPPH) of the aqueous extracts of the four *Sideritis* taxa from the Peloponnese were determined by colorimetric assays and the results are given in Table 2. The SR samples had the highest phenolic content, followed by SS, whereas the polyphenolic composition of SCC and SCP was 63% and 74% lower, respectively, in comparison to SR. The same pattern was observed for the total flavonoid content, where flavonoid composition was significantly higher in the SR samples, followed by SS, whereas SCC and SCP had the lowest flavonoid content (not significantly different between SCC and SCP). These results are in accordance with the LC/MS characterization in Section 2.2.

A very high and significant correlation was observed between total phenolic content and total flavonoid content (Table 3). These findings are consistent with the results of the LC/MS analysis, since the majority of the detected phenolics were flavonoids, especially in the SR extracts, which were the richest taxa in flavonoids.

Previous comparisons among *Sideritis* species also found that *S. scardica* samples had higher TPC than *S. raeseri* [5]. Overall, the values of total phenolics and total flavonoids herein were comparatively equal [40] or lower in comparison with previous studies [4,5,14,34,40] and the differences could be attributed to the extraction method, as well. The high content of total phenols and flavonoids of SR was depicted in its high antioxidant activity in both FRAP and DPPH assays. SS extract also exhibited high antioxidant properties but lower than SR in the case of FRAP. The IC_50_ values of SR and SS were much lower than those described in the study of Karapandzova et al. [41]. SCC displayed stronger antioxidant activities in comparison to SCP, but both were lower than SR and SS extracts. Finally, according to Pearson’s correlation matrix, FRAP was very highly and significantly correlated to phenolics and flavonoids, and DPPH was highly and significantly correlated to phenolics, flavonoids, and FRAP.

### 2.4. GC/MS Determination of Volatile Compounds in Petroleum Ether Extracts

The acidic pretreatment along with the ultrasound-assisted extraction with petroleum ether afforded significantly high yields for all studied samples; 1.92%, 0.70%, 2.58%, and 0.75% for SCP, SCC, SR, and SS, respectively. These high values can be justified by the fact that organic solvents also extract compounds such as fatty acids, esters, and hydrocarbons along with the essential oil (EO), in contrast to distillation, in which only volatile compounds are obtained. These results agree with earlier observations [16,42].

Seventy (70) compounds in total were identified in all extracts (Table 4). Specifically, 31, 50, 24, and 39 compounds were identified in SCP, SCC, SR, and SS, respectively. Among the identified compounds, only 13 were in common among all taxa: monoterpenes α-pinene, sabinene, β-pinene, o-cymene, sylvestrene, linalool and 1,8-cineole; sesquiterpenes α-copaene, β-bourbonene, β-elemene, β-caryophyllene, and caryophyllene oxide; and the alkane nonane. α-Pinene was found in relatively high percentages (18.26% to 20.40%) in almost all samples except for SR (2.64%). The percentage of the identified compounds varied among extracts, and it was more than 78% in all samples (82.88%, 78.75%, 79.97%, and 89.20% of the total extract content in SCP, SCC, SR, and SS, respectively). Not only the number but also the concentration of volatiles was the highest in SCC extract (41.31 mg/100 g plant material); it was approximately two-fold higher than the other samples.

**Figure 2:**
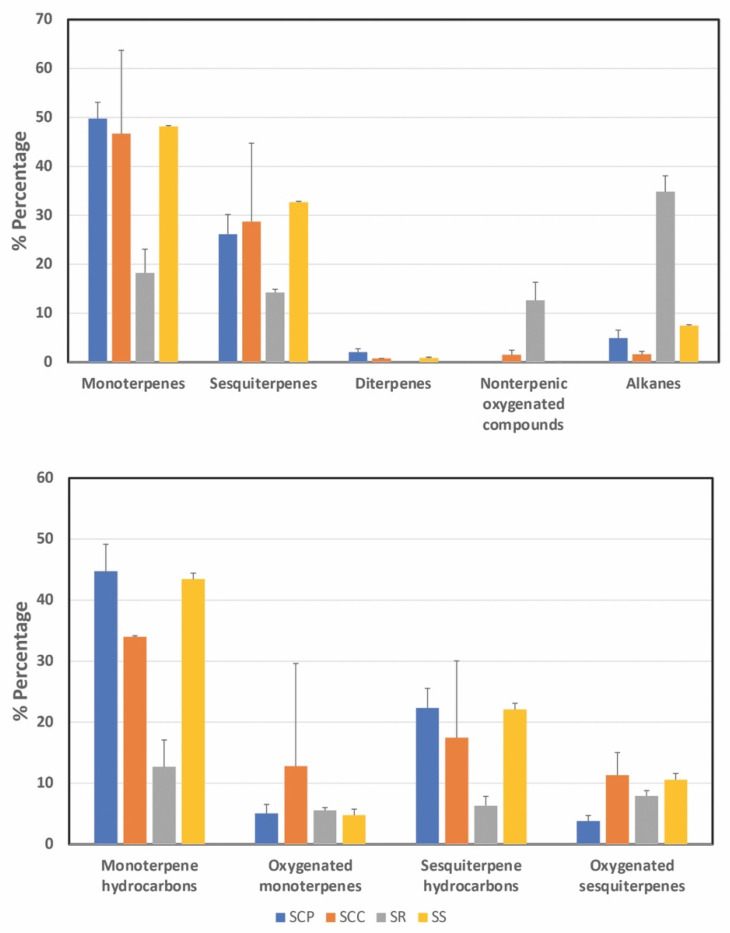
The content of each volatile category in the petroleum ether extracts of the four *Sideritis* taxa (2 biological samples per taxon, n = 2). The values of the percentage of peak areas (average ± standard deviation) are presented.

All samples were characterized by the presence of hydrocarbons that ranged from 53.85% in SR to 73.00% in SS, whereas oxygenated compounds were detected in lower percentages (9.94−41.09%). Monoterpene hydrocarbons were the dominant group in all samples (12.68−44.71%) (Figure 2). Oxygenated monoterpenes were in a high percentage in SCC (24.26%). SCC also contained the highest content of oxygenated sesquiterpenes. Diterpenes were detected only in SCC, SCP, and SS extracts and in very low amounts (<2.04%). The SR extract was characterized by the highest content in alkanes (34.84%) and non-terpenic oxygenated compounds (12.65%), outweighing monoterpenes (18.24%) and sesquiterpenes (14.24%).

In detail, in SCP the main components were the monoterpenes α-pinene (20.01%), β-pinene (7.73%), β-myrcene (5.86%), and sylvestrene (5.52%), as well as the sesquiterpenes bicyclogermacrene (6.01%) and germacrene D (5.28%) (Table 4). These results are quite consistent with previous studies concerning the high amounts of α-pinene and β-pinene [12,16].

In SCC extract, α-pinene (20.40%), linalool (13.90%), and β-pinene (6.47%) had the highest percentages. Other quantitatively important compounds were α-bisabolol (5.42%) and β-caryophyllene (4.86%). These results are consistent with previous studies [10,11,12].

In the study of Ntalli et al. [13], the major compounds in the essential oil of *S. clandestina* were β-pinene (8.3%), α-pinene (4.3%), and β-bisabolol (5.7%), as well as spathulenol (5.0%) (found in very low concentrations in our study). Comparing our results of *S. clandestina* to the literature [10,11,12,13], it is noteworthy to mention the absence of monoterpene sylvestrene, which was found in a significant amount (3.10%) in the present study, and also the consistency in the presence of α-pinene and β-pinene that characterizes the EO of most *Sideritis* species. It is interesting to note that the comparison of our SCC and SCP extracts revealed some qualitative and quantitative dissimilarities (Table 4); more compounds were detected in SCC, the total concentration of the volatiles was 77% higher, and the oxygenated monoterpenes were six-fold higher in comparison to SCP (10.07 over 1.48 mg/100 g plant material, respectively). Linalool and α−bisabolol were three-fold higher, whereas linalool acetate and epi-α-bisabolol were detected only in SCC in considerable concentrations.

As far as SR extract is concerned, the hydrocarbons *n*-decane (23.59%), nonane (11.25%), and hexadecanoic acid (11.09%) were the prevalent compounds (Table 4). Other quantitatively important volatiles were β-caryophyllene (4.28%), caryophyllene oxide (4.17%), α-bisabolol (3.75%), and eugenol (3.43%). According to Qazimi et al. [42], the *n*-hexane extracts of different populations of SR collected from Albania and North Macedonia showed up with a similar chemical load. The extracts consisted mainly of hydrocarbons, and the components *n*-decane, nonane, and hexadecanoic acid were present in different amounts. Comparing our results with previous studies using mainly hydrodistillation, significant dissimilarities in the chemical composition were observed. The analysis of SR Eos originating from Greece, North Macedonia, and Albania [45] indicated that they were rich in monoterpenes, whereas no alkanes were detected; α-pinene and β-pinene were found in very high amounts. The strong presence of monoterpenes is also supported in previous studies, and different compounds are reported as the main constituents [10,11,16,46,47]. On the contrary, Romanucci et al. [48] supported that most SR Eos consisted of sesquiterpenes, representing 60−70% of the total EO content, with bicyclogermacrene being the main sesquiterpene, along with spathulenol and β-caryophyllene, whereas monoterpenes were found in very low amounts. Kostadinova et al. [49] also supported that sesquiterpenes were in high concentrations, indicating germacrone, elemol acetate, and a-cadinol as the prevalent compounds. Β-Caryophyllene was also found in high concentrations in two Greek populations of cultivated SR [5]), whereas α-bisabolol and bicyclogermacrene were found in the population originating from Florina. Observations of great variety in essential-oil composition of close Greek SR populations (both from Kozani) have been made by Kloukina et al. [50]. This nonuniformity of the chemical composition among our results and those previously investigated could be attributed to the differences in the plant material used (we used leaves and flowers in a specific ratio and not stems), in addition to the extraction method of the volatiles (ultrasound-assisted extraction); in most other studies EOs were obtained with distillation. Furthermore, the genotypes, the site of collection, and the climatic conditions, which differ from year to year, could also be the reason for the qualitative and quantitative disparity [51].

The analysis of the SS extract revealed that the prevalent compounds were α-pinene (18.95%), nonane (7.48%), sabinene (6.44%), and β-pinene (6.04%) (Table 4). The high values of α-pinene and β-pinene agree with many previous studies [50,51,52,53,54]. However, some chemical variations were observed between our results and those of previous studies. According to Kouklina et al. [50], the analysis of the EO of SS cultivation from Chromio Kozani indicated m-camphorene (10.3%) as one of the main constituents, whereas the EO of an SS cultivation from Metamorfosis Kozani was rich in sesquiterpenes (45.9%) and not in monoterpenes (31.2%). Different chemical profiles have also been found among four different Greek cultivated populations of SS despite the same cultivation conditions [5]. Todorova et al. [55] reported that β-caryophyllene (18.8%) and nerolidol (12.1%) were the major compounds of an SS wild population originating from North Macedonia, along with β-farnesene (6.6%) and germacrene D (6.6%), whereas Kostadinova et al. [49] found that octadecenol and α-cadinol had a very strong presence in SS from North Macedonia. These discrepancies could be attributed to the different cultivation and environmental conditions such as altitude [50,54].

## 3. Materials and Methods

### 3.1. Plant Material

*Sideritis raeseri* Boiss. & Heldr. Subsp. *raeseri*, *Sideritis clandestina* subsp. *peloponnesiaca* (Boiss. & Heldr.) Baden, and *Sideritis clandestina* Bory & Chaub. Hayek subsp. *clandestina* were collected from the mountains Gaidourorahi (38°02′10.9″ N–22°13′08.9″ E), Dourdouvana (37°54′95.6″ N–22°15′03.7″ E), and Parnonas (37°12′55.5″ N–22°38′14.5″ E), whereas *Sideritis scardica* Griseb. Was collected from commercial cultivation. All plants were harvested during the summer (July) of 2016. Their aerial parts were air dried and voucher specimens were authenticated by Prof. Gregoris Iatrou (UPA 15685 for *S. raeseri* Gaidourorahi; UPA 15690 for *S. clandestina* subsp. *peloponnesiaca* Dourdouvana; UPA 31153 for *S. clandestina* subsp. *clandestina*).

### 3.2. Isolation of Polar Compounds from SCP Extracts

The dried and macerated aerial parts of SCP (943 g) were successively extracted with a total volume of 10 L petroleum ether, 7.5 L of ethyl acetate, and 7 L of methanol. In detail, the plant was extracted five times with each solvent at room temperature; the solvent was replaced every day with a fresh one and the extraction procedure lasted 15 days. The dried methanol extract was redissolved in methanol and processed with activated charcoal to remove chlorophylls. After two hours, the extract was decolorized and filtered through celite under vacuum. The filtrate was dried in a rotary evaporator, lyophilized, and weighed (11.44 g).

The aqueous fraction was further processed, and 0.451 g were submitted to solid-phase extraction (SPE). The cartridges used were octadecyl C_18_ (500 mg/3 mL) Strata™-X 33 µm from Phenomenex (Torrance, CA, USA). They were conditioned with 3 mL of methanol and equilibrated with 3 mL of water. The sample was diluted in 3 mL 1% acetic acid aqueous solution and the elution was performed with 1% acetic acid aqueous solution (A) and methanol (B). Gradient elution was performed as follows: 85% A, 65% A, 60% A, 50% A, and 0% A. The SPE procedure afforded Fraction 1 (210 mg), Fraction 2 (28.8 mg), Fraction 3 (16.4 mg), Fraction 4 (15.5 mg), and Fraction 5 (19.4 mg). Fractions 1–3 were subjected to RP-HPLC (HPLC-DAD 1260 Infinity II) from Agilent Technologies Inc. (Santa Clara, CA, USA) using a semi-preparative column C-18 (250 × 10 mm, 5 µm, 100 Å, Phenomenex), the flow rate was 1.5 mL/min, and the detection wavelengths were set at 210/254/280/330 nm. The HPLC isolation afforded compounds **1–7**.

Compounds **1** (2.4 mg, t_R_ = 15.18 min) and **2** (11.5 mg, t_R_ = 15.4 min) were isolated from Fraction 1. The mobile phase consisted of 0.2% formic acid aqueous solution (A) and acetonitrile (B). The gradient conditions were 95–65% A (0–30 min), 65–95% A (30–31 min), and 95% A (31–38 min). The concentration of the sample was 4 mg/mL and the injection volume was 70 μL.

Compounds **3** (0.6 mg, t_R_ = 15.85 min), **4** (1.2 mg, t_R_ = 19.65 min), and **5** (2.1 mg, t_R_ = 26.03 min) were isolated from Fraction 2. The mobile phase consisted of 0.2% formic acid aqueous solution (A) and methanol (B). The gradient conditions were as follows: 76% A (0–7 min), 76–60% A (7–22 min), 60% A (22–24 min), 60–76% A (24–26 min), and 76% A (26–31 min). The concentration of the sample was 4 mg/mL and the injection volume was 50 μL.

Compounds **6** (4.9 mg, t_R_ = 17.79) and **7** (1.2 mg, t_R_ = 28.13) were isolated from Fraction 3. The mobile phase consisted of 0.2% formic acid aqueous solution (A) and methanol (B). The gradient was as follows: 65% A (0–12 min), 65–45% A (12–29 min), 45–65% A (29–30 min), and 65% A (30–33 min). The concentration of the sample was 3.6 mg/mL and the injection volume was 50 µL.

Compounds **8** (5.5 mg, t_R_ = 7.42 min)–**9** (4.2 mg, t_R_ = 10.07 min) were isolated from ethyl acetate fraction. Specifically, 21.5 mg of the ethyl acetate fraction were loaded on a preparative TLC plate coated with silica gel GF_254_ (1 mm) from Macherey-Nagel GmbH & Co. KG (Dueren, Germany). The chromatogram was developed with EtOAc:CH_2_Cl_2_:HCOOH:AcOH:H_2_O (100:25:10:10:11, *v/v/v/v/v*) and examined under a UV lamp (254 and 365 nm). The fluorescent band was scraped out and extracted with methanol. The diluted mixture was centrifuged at 7000 rpm for 10 min and the supernatant was submitted to RP-HPLC on a semi-preparative column C-18. The detection wavelengths were set at 235, 268, and 330 nm. The mobile phase consisted of 0.2% formic acid aqueous solution (A) and methanol (B) and the gradient was as follows: 50–45% A (0–4 min), 45% A (4–9 min), 45–10% A (9–20 min), 10% A (20–23 min), 10–50% A (23–28 min), and 50% A (28–35 min) at a flow rate of 1.3 mg/mL.

NMR spectra (^1^H, ^13^C and 2D) were recorded on a Brucker 600 MHz (600 MHz for ^1^H, 150.9 MHz for ^13^C) Avance III HD Ascend TM spectrometer at the Center of Instrumental Analysis, University of Patras, Greece. The chemical shifts (δ) are reported in parts per million (ppm) and the residual solvent signal was used as an internal standard. HPLC-DAD 1260 Infinity II (Agilent Technologies Inc., Santa Clara, CA, USA) equipped with the software OpenLab 3.2 was used for the isolation of glycosides. Analysis of extracts and glycosides was performed in a single quadrupole LC/MS system of LC/MSD1260 Infinity II (Agilent Technologies, Inc.) equipped with OpenLab 3.2. software from Agilent Technologies Inc.

### 3.3. Determination of Polar and Volatile Metabolites in Different Sideritis Samples

#### 3.3.1. Extraction

The extraction protocol was described in our previous work [16], namely, ultrasound-assisted extraction with pretreatment in citrate buffer (UAE-A). In detail, three (3) grams of plant material (flowers and leaves in a ratio of 2:5, in small pieces) were incubated in 90 mL of 0.05 M citrate buffer pH 4.8 for 75 min in a water bath at 37 °C. The aqueous buffer was removed, and the plant material was further extracted with 80 mL of petroleum ether in an ultrasonic bath for 30 min. Each extraction step was performed twice. The aqueous buffer was collected, extracted with petroleum ether, dried (lyophilized), weighed, and stored at −20 °C until use. The organic layers were merged, washed with saturated sodium chloride solution, dried over anhydrous sodium sulphate, filtered, and concentrated under vacuum. The petroleum ether extracts were submitted to a nitrogen flow for about 10 min (until the petroleum ether was evaporated). After measuring their volume, they were stored in clear glass vials at −20 °C until use. The experiments were performed twice for each sample.

#### 3.3.2. Gas Chromatography–Mass Spectrometry (GC/MS)

Analysis was carried out in an Agilent 6890N GC apparatus coupled to an Agilent 5975 B mass spectrometer with a non-polar column HP-5MS (30 m × 0.25 mm × 0.25 μm film thickness) using electron impact (70 eV) ionization mode. Helium was used as a carrier gas and the flow rate for the HP-5MS column was 1 mL/min; the injected volume was 1 μL in splitless mode. The injector temperature was set to 280 °C and the source temp to 230 °C. Specifically, the initial oven temperature was 50 °C for 4 min, which was then ramped up 2 °C/min^−1^ to 92 °C, 4 °C/min^−1^ to 108 °C, 2 °C/min^−1^ to 130 °C, 1 °C/min ^−1^ to 150 °C, 5 °C/ min ^−1^ to 180 °C, and finally 15 °C/min^−1^ to 270 °C. Qualitative analysis was based on a comparison of the obtained MS spectra to literature data and of the retention indices (RI) on apolar columns [43,56]. RI values were calculated based on a series of linear alkanes, C8−C20 and C21−C40, using the Van den Dool and Kratz equation.

Quantification was performed as described in our previous published work [16]. Data were expressed in two forms: (a) as a percentage of peak area divided by that of the internal standard and (b) as milligrams of α-pinene equivalents per 100 g of dry plant material. The procedure yields were expressed in milliliters per 100 g of dry material since the extracts were all liquid at ambient temperature. Only the compounds for which the peak area exceeded 0.1% of total peak area are presented.

#### 3.3.3. Liquid Chromatography–Mass Spectrometry (LC/MS)

The single quadrupole LC/MS system of LC/MSD1260 Infinity II (Agilent Technologies, Inc., Santa Clara, CA, USA) was used in this study. The system was equipped with an ESI ion source and the mass range was *m*/*z* 100–1600 in full scan mode. Nitrogen was used as the gas for ionization. Working conditions were in ESI negative mode and separation was performed on a Poroshell 120 EC 18 column (4.6 × 100 mm, 2.7 µm) (Agilent Technologies, Inc.). The mobile phase consisted of 0.1% acetic acid in water (A) and methanol (B). Separation was carried out in 65 min under the following conditions: 0–8 min 15% B; 8–13 min 15–35% B; 13–18 min 35% B; 18–19 min 35–40% B; 19–27 min 40% B; 27–28 min 40–45% B; 28–35 min 45% B; 35–45 min 45–75% B; 45–55 min 75% B; 55–59 min 75–15% B; 59–65 min 15% B. The flow rate was 0.3 mL/min and the injection volume was 10 μL. The samples were prepared by diluting the dry aqueous extracts in methanol, and their final concentration was 10 mg/mL.

The identification of the compounds was based on comparison of their retention time and their obtained mass spectra to the literature. Furthermore, six reference compounds were used (melittoside, ajugoside, 7-*O*-acetyl-8-epi- loganic acid, chlorogenic acid, verbascoside, isoverbascoside) that were isolated in our laboratory. Rutin (HPLC > 99%) from Extrasynthese (Genay, France) was used for the quantification. The calibration curve was established for eight concentrations (2–16 µg/mL) through the equation y = 126832x + 81400 (R^2^ = 0.9951). The lower limit of quantitation (LLOQ) was 0.772 µg/mL and the lower limit of detection (LLOD) was 0.232 µg/mL.

The experiments were performed in duplicate for each sample and the results are expressed in mg rutin equivalents per 100 g of dry plant material. The procedure yields are expressed as g of lyophilized extract per 100 g of dry plant material.

### 3.4. Determination of Total Phenolics, Flavonoids Content, and Antioxidant Capacity (DPPH and FRAP) in Aqueous Buffer Extracts

Total phenolics, total flavonoids, and antioxidant capacity were measured in the aqueous extracts (in triplicate, twice) with methods adapted for 96-well plates, and the absorbance was measured in a UV/vis microplate reader (Sunrise, Tecan Austria) against blanks.

Total phenolic content was determined with the Folin–Ciocalteau reagent method [57,58] at 620 nm and is expressed as mg of gallic acid equivalents (GAE) per g of dry weight (D.W.) of plant material.

Total flavonoid content was determined with the aluminum chloride (AlCl_3_) method [59] at 405 nm and the results are expressed as mg of quercetin equivalents (QE) per g of D.W. of plant material. In detail, 75 µL of ddH_2_O were mixed with 5 µL of CH_3_COOH 1Μ, 16 µL extract or standard (quercetin) in ethanol 60% (*v/v*), 40 µL ethanol 95% (*v*/*v*), and 5 μL of AlCl_3_ 10% *w/v* incubated at RT for 45 min.

The antioxidant activity was evaluated with two different assays—ferric reducing antioxidant power (FRAP) and 2,2-diphenyl-1-picrylhydrazyl (DPPH) radical scavenging activity. The FRAP method [60] measures the ability of antioxidants to reduce the [Fe_III_(TPTZ)_2_]^3+^ to [Fe_II_(TPTZ)_2_]^2+^. In detail, 80 µL of FRAP solution (15 mL of a solution of 10 mM 2,4,6-tri(2-pyridyl)-s-triazine (TPTZ) in 40 mM HCl, 15 mL of 20 mM FeCl_3_.6H_2_O, and 75 mL of 300 mM acetate buffer solution, pH 3.6), were mixed with 55 µL of acetate buffer and 60 µL extract or standard (FeSO_4_.7H_2_O) and incubated at room temperature for 5 min. Absorbance was measured at 592 nm and the results are expressed as mmol Fe^2+^ per g of D.W.

In addition, the antioxidant activity was determined with the 2,2-diphenyl-1-picrylhydrazyl (DPPH) method [61,62], which measures the ability of antioxidants to scavenge the stable organic nitrogen radical DPPH•. Absorbance was measured at 540 nm and the results are expressed as IC_50_ g of D.W. of plant material. In detail, 195 µL of 0.1 mM DPPH (in methanol) were mixed with 5 µL of extract or BHT (butylated hydroxytoluene) used as standard (both diluted in methanol 50% *v/v*) and incubated at RT for 30 min.

### 3.5. Statistical Analysis

Averages and standard deviation were calculated using replicates from all samples. The data were tested for normality with the Shapiro–Wilk test (*a >* 0.01) and for homogeneity of variance with the Levene test. ANOVA and the post-hock Tukey test (*a* = 0.05) were performed at a significance level. Pearson’s correlation was performed for all variable pairs at a significance level of 95% (*a* = 0.05) and *r >* 0.90, *r >* 0.70, *r >* 0.50, and *r >* 0.30 were interpreted as very high, high, moderate, and low coefficients, respectively. SPSS version 25.0 (IBM Corp., Armonk, NY, USA) was used for data analysis.

## 4. Conclusions

This study contributes to the phytochemical characterization of *Sideritis clandestina* and suggests a methodology for the comparison of its volatile and polar metabolite composition to other *Sideritis* taxa. *Sideritis* plants have been used daily in Balkan countries as aromatic infusions/decoctions that not only provide sensory pleasure but also various health benefits, e.g., sedation and alleviation of common-cold symptoms. In the last decade, the pursuit of producing novel and unique herbal products of high added value amid the economic crisis has highlighted the potential of mountain tea, especially in the light of studies of its health benefits and of the European Medicines Agency’s approval for its use in traditional herbal medicinal products for the relief of cough associated with a cold and for the relief of mild stomach and gut discomfort. Beverages, cosmetics, and food supplements using *Sideritis* plant material were introduced to the market. In official documents and in the market, the term “mountain tea” or “ironwort” describes many species of *Sideritis* and, in many cases, there are no special references to the particular taxa used, as there is still a lack of studies describing the phytochemical characterization of each taxon. The amount of material collected from nature is controlled by agronomical decisions (no more than 2 kg per person are allowed), and uprooting is prohibited. Most of the mountain-tea material on the market comes from the cultivations of SR and SS in open and covered fields that have increased in recent years. *S. clandestina* taxa are endemic in the Peloponnese, not cultivated, and were recently assigned as critically endangered and threatened [63].

We herein describe the phytochemical characterization of *S. clandestina* subsp. *peloponnesiaca* (SCP) for the first time. Four iridoid glycosides, two phenolic glycosides, and three caffeoyl ester derivatives (chlorogenic acid, verbascoside, and isoverbascoside) were isolated from SCP for the first time; vanillic acid and salicylic glycosides are not common in *Sideritis* taxa. We herein optimized a methodology previously developed by us (ultrasound-assisted extraction of samples with petroleum ether after acidic pretreatment) to determine both volatile and polar metabolites using plant samples as small as 3 g. The isolation of those nine compounds from SCP greatly helped the LC/MS identification of polar metabolites in the aqueous extracts. Regarding aqueous extracts, *S. raeseri* and *S. scardica* had higher phenolic and flavonoid content and therefore antioxidant properties than the *S. clandestina* samples. SCC seemed to be the most aromatic taxon with almost twice the amount of volatiles as the others. In total, 27 polar and 70 volatile metabolites were determined. This methodology could be applied to chemotaxonomic studies after testing larger numbers of samples, the selection of genotypes during breeding efforts for the development of varieties, and certainly the authentication of new final herbal products.

## Figures and Tables

**Table 1:** List of polar metabolites and their concentrations (mg rutin equivalents/100 g of dry plant material, average values ± standard deviation) in *Sideritis clandestina* subsp. *peloponnesiaca* (SCP), *Sideritis clandestina* subsp. *clandestina* (SCC), *Sideritis raeseri* (SR), and *Sideritis scardica* (SS) aqueous extracts (2 biological samples, n = 2 for each sample).

a/a	t_R_ (min)	Components	M.W.	[M-H]^−^	Other Negative Ions	SCP	SCC	SR	SS
C1	6.96	Melittoside *	524	523	583 [M+Hac-H]^−^ 1070 [2M+Na-H]^−^	19.18 ± 2.95	15.52 ± 0.49	15.49 ± 3.01	n.d.
C2	12.00	Unknown	374	373	747 [2M-H]^−^ 769 [2M-2H+Na]^−^	n.d.	21.17 ± 4.89	n.d.	n.d.
C3	13.30	Unknown	374	373	747 [2M-H]^−^ 769 [2M-2H+Na]^−^	n.d.	28.57 ± 6.87	n.d.	n.d.
C4	17.65	Unknown	488	487	975 [2M-H]^−^	9.20 ± 5.28	29.19 ± 3.55	n.d.	29.66 ± 0.99
C5	18.20	Unknown	376	375	751 [2M-H]^−^ 773 [2M-2H+Na]^−^	n.d.	7.57 ± 5.71	n.d.	n.d.
C6	18.80	Chlorogenic acid *	354	353	191 (quinic acid) 375 [M+Na-2H]^−^ 707 [2M-H]^−^ 729 [2M-2H+Na]^−^	33.23 ± 7.45	56.81 ± 11.81	61.21 ± 2.57	65.65 ± 8.96
C7	22.85	β-Hydroxyverbascoside isomer [34]	640	639	661 [M+Na-2H]^−^	8.55 ± 3.05	11.45 ± 3.56	n.q.	16.80 ± 6.95
C8	23.65	β-Hydroxyverbascoside isomer [34]	640	639	661 [M+Na-2H]^−^	9.20 ± 4.14	15.48 ± 3.81	9.78 ± 0.32	19.34 ± 6.79
C9	24.81	7-*O*-Acetyl-8-epi- loganic acid *	418	417	835 [2M-H]^−^ 857 [2M+Na-2H]^−^	7.29 ± 4.68	n.d.	11.76 ± 0.17	n.d.
C10	25.57	Ajugoside *	390		449 [M+Hac-H] 779 [2M-H]^−^	n.q.	n.d.	27.62 ± 3.84	n.d.
C11	28.60	Forsythoside B or Lavandulofolioside [34,35]	756	755	377 [M-2H]^−2^ 1512 [2M-H]^−^	n.d.	n.q.	29.42 ± 3.56	n.d.
C12	30.00	All-Glc-ISC [8]	610	609	1220 [2M-H]^−^	n.d.	n.d.	n.d.	25.46 ± 1.34
C13	30.30	Verbascoside *	624	623	311 [M-2H]^−2^ 1248 [2M-H]^−^	n.q.	23.31 ± 10.52	52.53 ± 5.22	39.05 ± 2.31
C14	31.80	All-Glc-HYP [8]	626	625	1251 [2M-H]^−^	8.59 ± 0.94	n.d.	18.13 ± 4.01	28.79 ± 1.44
C15 #	34.78	Allysonoside/Forsythoside B or lavandulofolioside [6,8,22,37]	770/756	769/755		n.d.	n.d.	11.58 ± 0.63	n.d.
C16 #	35.60	Leucoseptoside isomer/ Isoverbascoside * [8,37]	638/624	637/623		n.d.	n.d.	n.q.	7.57 ± 1.39
C17	36.40	AcO-All-Glc-ISC or AcO-All-Glc-LUT [8,37]	652	652	325 [M-2H]^−2^	n.d.	n.d.	n.d.	14.82 ± 0.82
C18	36.90	All-Glc-LUT [8]	610	609	1219 [2M-H]^−^	15.86 ± 4.56	18.29 ± 0.44	16.36 ± 5.71	20.72 ± 3.95
C19	37.00	AcO-All-Glc-HYP [6,8]	668	667		n.d.	n.d.	9.44 ± 2.08	7.51 ± 0.04
C20	38.50	All-Glc-HYP-Me [6,8]	640	639	1279 [2M-H]^−^	11.82 ± 1.60	10.33 ± 1.43	15.29 ± 3.24	11.28 ± 1.69
C21	39.30	AcO-All-Glc-HYP [6]	668	667	1335 [2M-H]^−^	n.q.	n.d.	28.71 ± 0.59	38.55 ± 1.21
C22	44.50	AcO-All-Glc-ISC or AcO-All-Glc-LUT [8,35,37]	652	651	1303 [2M-H]^−^	9.07 ± 3.21	11.42 ± 1.78	16.25 ± 0.29	13.09 ± 0.58
C23	44.90	AcO-All-Glc-HYP-Me [35,37]	682	681	1364 [2M-H]^−^	7.25 ± 0.66	7.16 ± 0.88	12.85 ± 0.55	n.q.
C24	45.80	AcO-All-Glc-HYP [8,35,37]	668	667	1336 [2M-H]^−^	n.q.	n.d.	11.71 ± 0.65	n.q.
C25	48.45	(AcO)_2_-All-Glc-HYP [8,35,36]	710	709	1419 [2M-H]^−^	n.q.	n.d.	11.90 ± 2.00	10.60 ± 0.29
C26	49.09	AcO-All-Glc-ISC-Me [8,35,36]	666	665	1332 [2M-H]^−^ 1354 [2M-2H+Na]^−^	n.d.	6.29 ± 0.44	n.d.	n.d.
C27 #	49.95	(AcO)_2_-All-Glc-ISC/ (AcO)_2_-All-Glc-HYP-Me [8,35,37]	694/724	693/723	733.7 [M+Hac-H]^−^ 1388 [2M-H]^−^ 1448 [2M-H]^−^	6.30 ± 1.12	n.q.	13.11 ± 1.68	n.q.
		Total				145.55	262.56	373.12	348.90

* Reference compounds have been used for the identification. # Those peaks were a mixture of two compounds that co-eluted. Abbreviations: n.q.: not quantified, n.d.: not detected, AcO: O-acetyl, All: allosyl, Glc: glucoside, HYP: hypolaetin, ISC: isoscutellarein, LUT: luteolin, Me: methyl, Hac: acetic acid.

**Table 2:** Total phenolics (TPC), total flavonoids (TFC), and antioxidant properties (DPPH radical scavenging activity and ferric ion reducing power (FRAP)) of the *Sideritis* aqueous extracts. The results are expressed as average values ± standard deviation (SD) of triplicate analysis (n = 2 biological samples per taxon). Significant differences among group means were determined by ANOVA and post-hock Tukey’s test (a = 0.05) and are indicated with different letters in superscript. The same letter indicates no statistically significant differences across rows at the confidence level of 95%.

	TPC (mg GAE/g) ^1^	TFC (mg QE/g) ^2^	FRAP Assay (mmol Fe^II^/g) ^3^	DPPH Assay IC_50_ (mg/mL) ^4^
**SCP**	3.19 ± 0.44 ^d^	2.40 ± 0.41 ^c^	19.48 ± 0.88 ^d^	5.44 ± 0.63 ^a^
**SCC**	4.62 ± 0.67 ^c^	2.94 ± 0.43 ^c^	39.94 ± 5.82 ^c^	3.11 ± 0.59 ^b^
**SR**	12.38 ± 1.23 ^a^	10.67 ± 1.20 ^a^	94.89 ± 7.41 ^a^	1.79 ± 0.19 ^c^
**SS**	9.53 ± 1.69 ^b^	9.18 ± 1.43 ^b^	78.41 ± 11.09 ^b^	1.73 ± 0.31 ^c^

^1^ Expressed as mg of gallic acid (GAE) per g of dry plant material. ^2^ Expressed as mg of quercetin (QE) per g of dry plant material. ^3^ Expressed as mmol Fe^II^ per g of dry plant material. ^4^ Expressed as IC_50_ values corresponding to the aqueous extract concentration (mg of dry plant material/mL) causing 50% inhibition of DPPH radical.

**Table 3:** Pearson’s correlation matrix of all values of total phenolic content (TPC), total flavonoid content (TFC), FRAP values, and the IC_50_ DPPH.

	TPC	TFC	FRAP	DPPH IC_50_
**TPC**	1			
**TFC**	0.982 **	1		
**FRAP**	0.987 **	0.981 **	1	
**DPPH IC_50_**	−0.853 **	−0.849 **	−0.919 **	1

** *p* < 0.01.

**Table 4:** Percentages and concentrations of volatile components identified in petroleum ether extracts of *Sideritis clandestina* subsp. *peloponnesiaca* (SCP), *Sideritis clandestina* subsp. *clandestina* (SCC), *Sideritis raeseri* subsp. *raeseri* (SR), and *Sideritis scardica* (SS) (2 biological samples, n = 2 for each sample). The percentages are expressed as normalized peak areas (peak area of component/peak area of internal standard). Concentrations are expressed as mg α-pinene equivalents/100 g dry plant material (average ± standard deviation).

				SCP		SCC		SR		SS	
No	RI _cal_ ^1^	RI _lit_ ^2^	Components	%	mg/100 g	%	mg/100 g	%	mg/100 g	%	mg/100 g
1	901	900	Nonane	4.26	1.11 ± 0.35	1.17	0.52 ± 0.03 *	11.25	2.13 ± 0.03	7.48	1.31 ± 0.17
2	923	924	α-Thujene	0.74	0.32 ± 0.02	0.29	0.17 ± 0.03	n.d.	n.d.	n.q.	n.q.
3	929	932	α-Pinene	20.01	4.70 ± 0.29	20.40	8.31 ± 0.57	2.64	0.54 ± 0.15	18.26	3.18 ± 0.42 *
4	969	969	Sabinene	1.61	0.51 ± 0.06	0.73	0.35 ± 0.07	n.q.	n.q.	6.44	1.13 ± 0.06
5	971	974	β-Pinene	7.73	1.91 ± 0.21	6.47	2.68 ± 0.02	3.89	0.78 ± 0.26	6.04	1.06 ± 0.01
6	979	974	1-Octen-3-ol	n.d.	n.d.	0.43	0.23 ± 0.01	n.d.	n.d.	n.d.	n.d.
7	991	988	β-Myrcene	5.86	1.48 ± 0.25	n.d.	n.d.	n.d.	n.d.	2.28	0.41 ± 0.08
8	1000	1000	*n*-Decane	0.69	0.30 ± 0.07	n.d.	n.d.	23.59	4.42 ± 0.32	n.d.	n.d.
9	1002	1002	α-Phellandrene	0.74	0.31 ± 0.02	1.02	0.47 ± 0.17	n.d.	n.d.	n.d.	n.d.
10	1007	1008	3-Carene	0.87	0.34 ± 0.05	1.44	0.64 ± 0.18	0.56	0.15 ± 0.01 *	n.d.	n.d.
11	1014	1014	α-Terpinene	0.57	0.28 ± 0.05	n.d.	n.d.	n.d.	n.d.	1.69	0.30 ± 0.07
12	1022	1022	*o*-Cymene	0.94	0.36 ± 0.09	0.37	0.20 ± 0.05	2.83	0.58 ± 0.38	1.75	0.31 ± 0.16
13	1025	1025	Sylvestrene	5.52	1.41 ± 0.24	3.10	1.31 ± 0.41	1.39	0.31 ± 0.06	4.76	0.85 ± 0.29
14	1027	1026	1,8-Cineole	1.30	0.46 ± 0.33	0.58	0.28 ± 0.17	n.q.	n.q.	n.q.	n.q.
15	1046	1053	*trans*-Decahydro-naphthalene	0.31	0.22 ± 0.03	n.d.	n.d.	1.65	0.35 ± 0.03	1.50	0.27 ± 0.01
16	1056	1054	γ-Terpinene	n.d.	n.d.	n.d.	n.d.	n.q.	n.q.	0.72	0.14 ± 0.01
17	1085	1086	Terpinolene	n.d.	n.d.	0.17	0.12 ± 0.01 *	n.d.	n.d.	n.d.	n.d.
18	1101	1095	Linalool	3.73	1.02 ± 0.24	13.90	5.57 ± 0.11 *	n.q.	n.q.	3.59	0.63 ± 0.14
19	1138	1141	Camphor	n.d.	n.d.	2.68	1.12 ± 0.05 *	n.d.	n.d.	n.d.	n.d.
20	1151	1155	Isoborneol	n.d.	n.d.	0.41	0.22 ± 0.01 *	n.d.	n.d.	n.d.	n.d.
21	1160	1165	Borneol	n.d.	n.d.	1.48	0.64 ± 0.02 *	n.d.	n.d.	n.d.	n.d.
22	1174	1174	Terpinen-4-ol	n.d.	n.d.	0.72	0.34 ± 0.01 *	n.d.	n.d.	n.d.	n.d.
23	1187	1186	α-Terpineol	n.d.	n.d.	0.72	0.34 ± 0.06	n.d.	n.d.	0.69	0.13 ± 0.01
24	1200	1200	*n*-Dodecane	n.d.	n.d.	0.45	0.24 ± 0.09	n.d.	n.d.	n.d.	n.d.
25	1260	1254	Linalool acetate	n.d.	n.d.	3.76	1.55 ± 0.15 *	n.d.	n.d.	n.d.	n.d.
26	1332 ^#^	1324	Bicycloelemene	3.49	0.95 ± 0.22	0.71	0.34 ± 0.16	n.d.	n.d.	n.d.	n.d.
27	1355	1356	Eugenol	n.d.	n.d.	n.d.	n.d.	3.43	0.69 ± 0.15	0.47	0.10 ± 0.03
28	1360	1369	Cyclosativene	n.d.	n.d.	n.d.	n.d.	n.d.	n.d.	0.75	0.15 ± 0.02
29	1370	1370	α-Copaene	n.q.	n.q.	0.93	0.44 ± 0.08 *	n.q.	n.q.	4.05	0.72 ± 0.14
30	1378	1387	β-Bourbonene	0.62	0.29 ± 0.06	0.33	0.19 ± 0.01 *	0.91	0.22 ± 0.01 *	1.60	0.30 ± 0.12
31	1388	1389	β-Elemene	n.q.	n.q.	0.22	0.14 ± 0.01 *	n.q.	n.q.	0.32	0.07 ± 0.01 *
32	1413	1417	β-Caryophyllene	3.82	1.03 ± 0.22	4.86	2.04 ± 1.12	4.28	0.84 ± 0.08	4.33	0.77 ± 0.24
33	1423	1430	β-Copaene	n.d.	n.d.	n.d.	n.d.	n.d.	n.d.	0.39	0.08 ± 0.02
34	1444	1451	*trans*-Muurola,3,5-diene	n.d.	n.d.	0.25	0.15 ± 0.01 *	n.d.	n.d.	n.d.	n.d.
35	1446	1452	α-Humulene	n.d.	n.d.	0.28	0.17 ± 0.02 *	n.d.	n.d.	n.d.	n.d.
36	1456	1456	β-Farnesene	n.d.	n.d.	0.92	0.43 ± 0.03 ^§,^ *	n.d.	n.d.	1.10	0.21 ± 0.15
37	1457	1464	α-Acoradiene	0.54	0.27 ± 0.04	n.d.	^§,^ *	n.d.	n.d.	n.d.	n.d.
38	1461 ^#^	1464	9-epi-(E)-β-Caryophyllene	n.d.	n.d.	3.12	1.33 ± 0.57	n.d.	n.d.	n.d.	n.d.
39	1474	1478	γ-Muurolene	n.d.	n.d.	1.89	0.83 ± 0.97	n.d.	n.d.	n.d.	n.d.
40	1474	1480	Germacrene D	5.28	1.37 ± 0.28	n.d.	n.d.	1.35	0.30 ± 0.06	2.60	0.47 ± 0.15
41	1477	1481	γ-Curcumene	0.71	0.30 ± 0.02	0.38	0.21 ± 0.01 *	n.d.	n.d.	n.d.	n.d.
42	1480	1480	a-Curcumene	0.87	0.35 ± 0.06	0.44	0.23 ± 0.03 *	n.d.	n.d.	0.64	0.12 ± 0.07
43	1490	1493	epi-Cubebol	n.d.	n.d.	n.d.	n.d.	n.d.	n.d.	0.90	0.17 ± 0.01
44	1490	1500	Bicyclogermacrene	6.01	1.54 ± 0.54	1.35	0.61 ± 0.33	n.d.	n.d.	n.q.	n.q.
45	1493	1493	α-Zingiberene	1.03	0.38 ± 0.06	0.70	0.34 ± 0.02 *	n.d.	n.d.	n.d.	n.d.
46	1506	1505	β-Bisabolene	n.d.	n.d.	1.55	0.68 ± 0.16	0.48	0.14 ± 0.01 *	n.d.	n.d.
47	1509	1514	β-Curcumene	n.d.	n.d.	0.60	0.30 ± 0.03 *	n.d.	n.d.	n.d.	n.d.
48	1510	1514	Cubebol	n.d.	n.d.	0.62	0.31 ± 0.04 *	n.d.	n.d.	1.23	0.23 ± 0.03
49	1518	1528	*cis*-Calamenene	n.d.	n.d.	0.93	0.43 ± 0.03 *	n.d.	n.d.	1.39	0.26 ± 0.10
50	1518	1522	δ-Cadinene	n.d.	n.d.	0.38	0.21 ± 0.01 *	n.d.	n.d.	4.75	0.84 ± 0.08
51	1519 ^#^	1522	Dihydroactinidiolide	n.d.	n.d.	n.d.	n.d.	2.13	0.44 ± 0.03	n.d.	n.d.
52	1526	1534	*trans*-Cadina-1(2)4-diene	n.d.	n.d.	1.58	0.70 ± 0.01 *	n.d.	n.d.	0.34	0.08 ± 0.01 *
53	1543	1549	Elemol	n.d.	n.d.	n.d.	n.d.	n.d.	n.d.	0.95	0.18 ± 0.11
54	1568	1578	Spathulenol	2.06	0.62 ± 0.12	0.31	0.18 ± 0.08	n.d.	n.d.	n.d.	n.d.
55	1572	1583	Caryophyllene oxide	n.q.	n.q.	1.29	0.58 ± 0.07	4.17	0.82 ± 0.13	2.63	0.48 ± 0.12
56	1583	1590	Globulol	n.d.	n.d.	0.17	0.12 ± 0.02 *	n.d.	n.d.	1.74	0.31 ± 0.10
57	1621	1628	1-epi-Cubenol	n.d.	n.d.	n.d.	n.d.	n.d.	n.d.	0.72	0.14 ± 0.03
58	1621	1631	Muurola-4,10(14)-dien-1β-οl	n.d.	n.d.	n.d.	n.d.	n.d.	n.d.	0.57	0.11 ± 0.02
59	1634	1642	α-epi-Muurolol	n.d.	n.d.	n.d.	n.d.	n.d.	n.d.	0.37	0.08 ± 0.01 *
60	1654	1666/1668	14-Hydroxy-(Z)-caryophyllene/14-Hydroxy-9-*epi*-(E)-caryophyllene	n.d.	n.d.	0.17	0.12 ± 0.01 *	n.d.	n.d.	n.d.	n.d.
61	1660	1668	*trans*-Calamenen-10-ol	n.d.	n.d.	n.d.	n.d.	n.d.	n.d.	0.81	0.16 ± 0.04
62	1660	1675	Valeranone	n.d.	n.d.	0.78	0.36 ± 0.02 *	n.d.	n.d.	n.d.	n.d.
63	1677	1683	α-*epi*-Bisabolol	n.d.	n.d.	3.42	1.45 ± 0.19	n.d.	n.d.	n.d.	n.d.
64	1677	1685	Germacra-4(15),5,10(14)-trien-1-a-ol	n.d.	n.d.	n.d.	n.d.	n.d.	n.d.	0.67	0.13 ± 0.04
65	1678	1685	α-Bisabolol	1.75	0.56 ± 0.19	5.42	2.26 ± 0.66	3.75	0.74 ± 0.02	n.d.	n.d.
66	1754	1759	Benzyl benzoate	n.d.	n.d.	n.d.	n.d.	1.56	0.34 ± 0.01	n.d.	n.d.
67	1975	1997	Kaur-15-ene	1.26	0.42 ± 0.08	0.74	0.35 ± 0.01	n.d.	n.d.	n.d.	n.d.
68	1979	1960	Hexadecanoic acid	n.d.	n.d.	n.d.	n.d.	11.09	2.09 ± 0.58	n.d.	n.d.
69	1995	1987/2009	Manool oxide/ 13-*epi*-Manool oxide	1.10	0.40 ± 0.07	n.d.	n.d.	n.d.	n.d.	0.86	0.16 ± 0.01
70	2066 ^#^	2060	Oleyl alcohol	n.d.	n.d.	1.10	0.51 ± 0.40	n.d.	n.d.	n.d.	n.d.
			Total	82.88	23.05 ± 2.78	78.75	41.31 ± 0.79	79.97	15.64 ± 0.41	89.20	16.01 ± 0.67
			Number of compounds	31		50		24		38	

Note: n.q.: not quantified, n.d.: not detected. ^1^ Retention index on HP-5MS (non-polar column). ^2^ Literature retention index on the non-polar column as reported in [43] except those with ^#^, which are reported in König et al. [44]. * These compounds were detected only in one biological sample. ^§^ These compounds did not separate, and the values refer to both compounds.

## Data Availability

Data will be made available on request.

## Supplementary Materials

The following supporting information can be downloaded at: https://www.mdpi.com/article/10.3390/molecules27217613/s1, Figure S1. ^1^H-NMR spectrum of monomelittoside (**1**) (^1^H-NMR:600 MHz; D_2_O). Figure S2. MS spectrum of monomelittoside (**1**) in negative mode. The major ions were 407 [M+FA-H]^−^, 361 [M-H]^−^, and 723 [2M-H]^−^. Figure S3. UV-vis spectrum of monomelittoside (**1**) showing a λ_max_ of 210 nm. Figure S4. ^1^H-NMR spectrum of melittoside (**2**) (^1^H-NMR:600 MHz; D_2_O). Figure S5. ^13^C-NMR spectrum of melittoside (**2**) (^13^C-NMR:150 MHz; D_2_O). Figure S6. MS spectrum of melittoside (**2**) in negative mode. The major ions were 523 [M-H]^−^, 583 [M+Hac-H]^−^, and 1047 [2M-H]^−^. Figure S7. UV-vis spectrum of melittoside (**2**) showing a maximum absorbance at 210 nm. Figure S8. ^1^H-NMR spectrum of vanillic acid glucoside (**3**) (^1^H-NMR:600 MHz; D_2_O). Figure S9. MS spectrum of vanillic acid glucoside (**3**) in negative mode. The major ions were 659 [2M-H]^−^ and 329 [M-H]^−^. Figure S10. UV spectrum of vanillic acid glucoside (**3**). Maximum absorbance at 210, 254, and 290 nm. Figure S11. ^1^H-NMR spectrum of ajugoside (**4**) (^1^H-NMR:600 MHz; D_2_O). Figure S12. MS spectrum of ajugoside (**4**) in negative mode. The major ions were 449 [M+Hac-H]^−^ and 779 [2M-H]^−^. Figure S13. UV-vis spectrum of ajugoside (**4**). Maximum absorbance at 200 nm. Figure S14. ^1^H-NMR spectrum of salicylic acid glucoside (**5**) (^1^H-NMR:600 MHz; D2O). Figure S15. MS spectrum of salicylic acid glucoside (**5**) in negative mode. The major ions of 299 and 599 correspond to [Μ-H]^−^ and [2M-H]^−^, respectively. Figure S16. UV-vis spectrum of salicylic acid glucoside (**5**) having maximum absorbance at 230 and 286 nm. Figure S17 ^1^H-NMR spectrum of 7-O-acetyl-8-epi-loganic acid (**6**) (^1^H-NMR:600 MHz; D_2_O). Figure S18. MS spectrum of 7-O-acetyl-8-epi-loganic acid (**6**) in negative mode. The major ions were 417 [Μ-H]^−^ and 835 [2M-H]^−^. Figure S19. UV spectrum of 7-O-acetyl-8-epi-loganic acid (**6**) with maximum absorbance at 236 nm. Figure S20. ^1^H-NMR spectrum of chlorogenic acid (**7**) (^1^H-NMR:600 MHz; CD_3_OD). Figure S21. MS spectrum of chlorogenic acid (**7**) in negative mode. The major ions of 707 and 353 correspond to [2M-H]^−^ and [M-H]^−^, respectively, whereas the ion of m/z = 191 is a fragment corresponding to quinic acid. Figure S22. UV-vis spectrum of chlorogenic acid (**7**) showing maximum absorbance at 330 nm. Figure S23. ^1^H-NMR spectrum of verbascoside (**8**) (^1^H-NMR:600 MHz; D_2_O). Figure S24. ^13^C-NMR spectrum of verbascoside (**8**) (^13^C-NMR:150 MHz; D2O). Figure S25. HSQC-NMR spectrum of verbascoside (**8**) (D_2_O). Figure S26. HMBC-NMR spectrum of verbascoside (**8**) (D_2_O). Figure S27. COSY-NMR spectrum of verbascoside (**8**) (D_2_O). Figure S28. ROESY- NMR spectrum of verbascoside (**8**) (D_2_O). Figure S29. MS spectrum of verbascoside (**8**) in negative mode with the sole ion of 623 [M-H]^−^. Figure S30. UV spectrum of verbascoside (**8**) showing maximum absorbance at 210 and 330 nm. Figure S31. ^1^H-NMR spectrum of isoverbascoside (**9**) (^1^H-NMR:600 MHz; D_2_O). Figure S32. ^13^C-NMR spectrum of isoverbascoside (**9**) (^13^C-NMR:150 MHz; D_2_O). Figure S33. HSQC-NMR spectrum of isoverbascoside (**9**) (D_2_O). Figure S34. HMBC-NMR spectrum of isoverbascoside (**9**) (D_2_O). Figure S35. COSY-NMR spectrum of isoverbascoside (**9**) (D_2_O). Figure S36. ROESY- NMR spectrum of isoverbascoside (**9**) (D_2_O). Figure S37. MS spectrum of isoverbascoside (**9**) in negative mode with the main ion of 623 [M-H]^−^. Figure S38. UV-vis spectrum of isoverbascoside (**9**) showing absorbance maxima at 210 and 330 nm. Figure S39. Total ion chromatogram (TIC) in negative mode of *Sideritis clandestina* subsp. *peloponnesiaca* (SCP) aqueous extract. Table S1. ^1^H-NMR (600 MHz) data of monomelittoside (**1**) in D_2_O. Table S2. ^1^H-NMR (600 MHz) and ^13^C-NMR (150 MHz) data of melittoside (**2**) in D_2_O. Table S3. ^1^H-NMR (600 MHz) data of vanillic acid glucoside (**3**) in D_2_O. Table S4. ^1^H-NMR (600 MHz) data of ajugoside (**4**) in D_2_O. Table S5. ^1^H-NMR data of salicylic acid glucoside (**5**) (D_2_O). Table S6. ^1^H-NMR data of 7-O-acetyl-8-epi-loganic acid (**6**) (D_2_O). Table S7. ^1^H-NMR data of chlorogenic acid (**7**) (CD_3_OD). Table S8. ^1^H-NMR, ^13^C-NMR and APT-NMR data of verbascoside (**8**) (D_2_O). Table S9. ^1^H-NMR, ^13^C-NMR and APT-NMR data of isoverbascoside (**9**) (D_2_O).
