# Supplementary material for: Characterization of Sideritis clandestina subsp. peloponnesiaca Polar Glycosides and Phytochemical Comparison to Other Mountain Tea Populations
Source: Molecules. 2022 Nov 6;27(21):7613. doi: 10.3390/molecules27217613 (PMC9658581; doi:10.3390/molecules27217613)
Supplement: Supplementary file 1 [file molecules-27-07613-s001.zip › molecules-1966624-supplementary.pdf]

SUPPLEMENTARY INFORMATION

Characterization of *Sideritis clandestina* subsp. *peloponnesiaca* polar glycosides and phytochemical comparison to other mountain tea populations

Virginia D. Dimaki [a], Konstantina Zeliou [a], Fotini Nakka [a], Michaela Stavreli [a], Ioannis Bakratsas [a], Ligeri Papaioannou [a, b], Gregoris Iatrou [b], Fotini N. Lamari [a, *]

[a] Laboratory of Pharmacognosy & Chemistry of Natural Products, Department of Pharmacy, University of Patras, Rio University Campus, 26504 Patras, Greece

[b] Division of Plant Biology, Department of Biology, University of Patras, Rio University Campus, 26504 Patras, Greece

* Corresponding author. E-mail: flam@upatras.gr. Tel.: +30 2610962335.

Part A. Structure elucidation of isolated compounds

Part B. Other Data

Part A. Structure elucidation of isolated compounds

Monomelittoside (1)

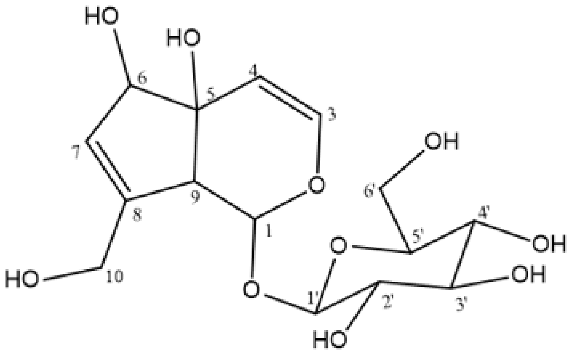

Table S1. ^{1}H-NMR (600 MHz) data of monomelittoside (**1**) in D_2O.

Position	δ_H (ppm, *J* in Hz)
1	5.73 (d, *J*=2.6)
3	6.37 (d, *J*=6.4)
4	5.07 (dd, *J*=6.3, 1.2)
5	-
6	4.24 (brs)
7	5.89 (brs)
8	-
9	3.22 (brs)
10	4.26 (s)
1'	*
2' 3' 4' 5'	3.55-3.42 (m) **
6a'	3.96 (dd, *J*=12.4, 2.1)
6b'	3.76 (dd, *J*=12.4, 6.0)

*overlapped by the signal of the solvent **assignments overlapped

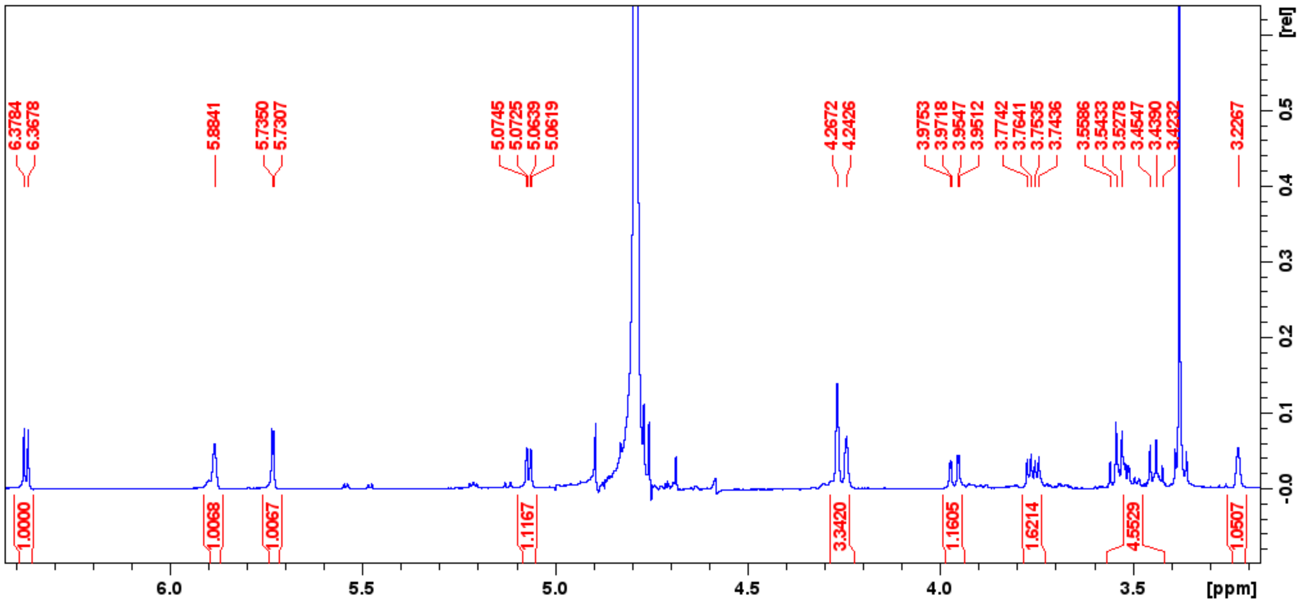

Figure S1. ^{1}H-NMR spectrum of monomelittoside (**1**) (^{1}H-NMR:600 MHz; D_2O)

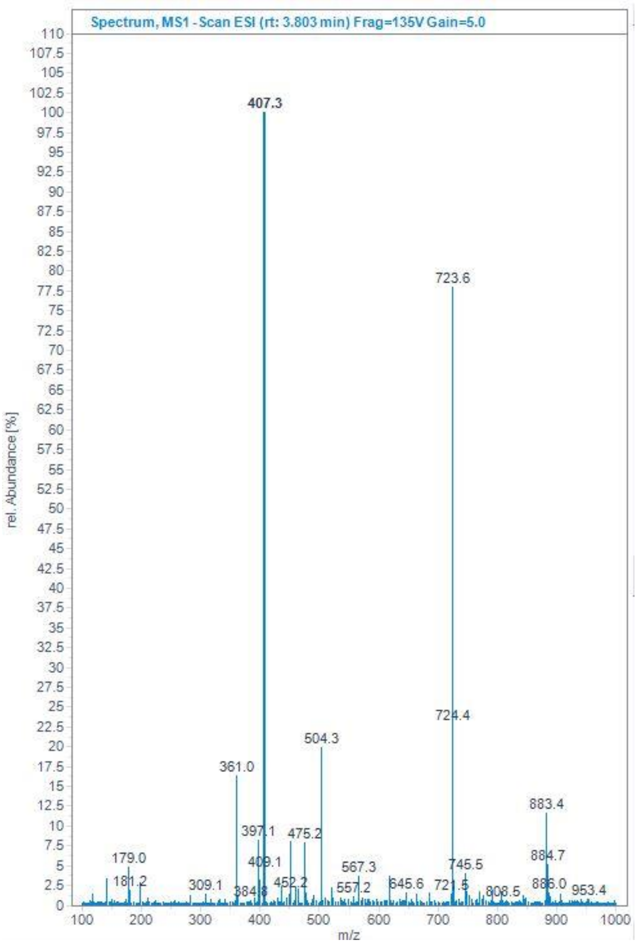

Figure S2. MS spectrum of monomelittoside (**1**) in negative mode. The major ions were 407 [M+FA-H]$^{-}$, 361 [M-H]$^{-}$ and 723 [2M-H]$^{-}$.

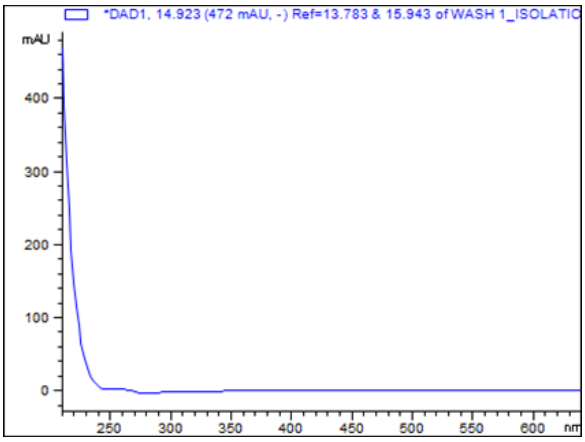

Figure S3. UV-vis spectrum of monomelittoside (**1**) showing a λ_{max} of 210 nm.

Melittoside (2)

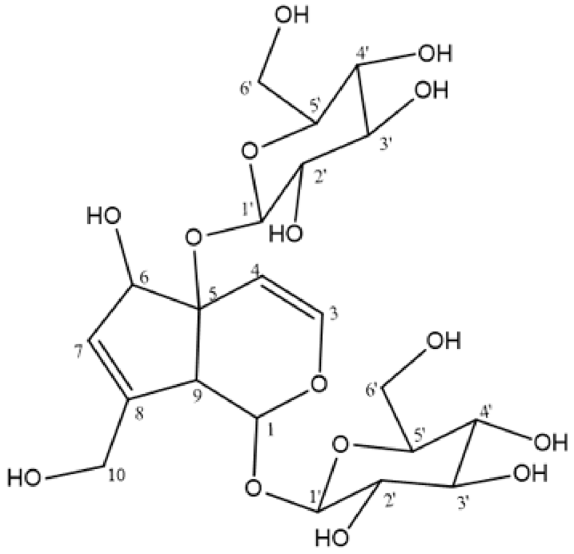

Table S2. ^{1}H-NMR (600 MHz) and ^{13}C-NMR (150 MHz) data of melittoside (**2**) in D_2O

Position	δ_H (ppm, *J* in Hz)	δ_C (ppm)
1	5.48 (d, *J*=5.4)	95.2
3	6.55 (d, *J*=6.4)	143.4
4	5.21 (d, *J*=6.4)	104.7
5	-	80.7
6	4.63 (s)	79.6
7	5.90 (brs)	127.6
8	-	144.6
9	3.56-3.35 (m)**	50.6
10	4.33-4.25 (m)	59.6
1'	*	98.0
2'	3.56-3.35 (m)**	72.8
3'	3.56-3.35 (m)**	75.6
4'	3.56-3.35 (m)**	69.3
5'	3.56-3.35 (m)**	75.5
6a' 6b'	3.94-3.90 (m)	60.5
1"	*	97.8
2"	3.56-3.35 (m)**	73.1
3"	3.56-3.35 (m)**	76.2
4"	3.56-3.35 (m)**	69.4
5"	3.56-3.35 (m)**	75.7
6a" 6b"	3.76 (dd, *J*=12.4, 5.3)	60.6

*Overlapped by the signal of the solvent **assignments overlapped

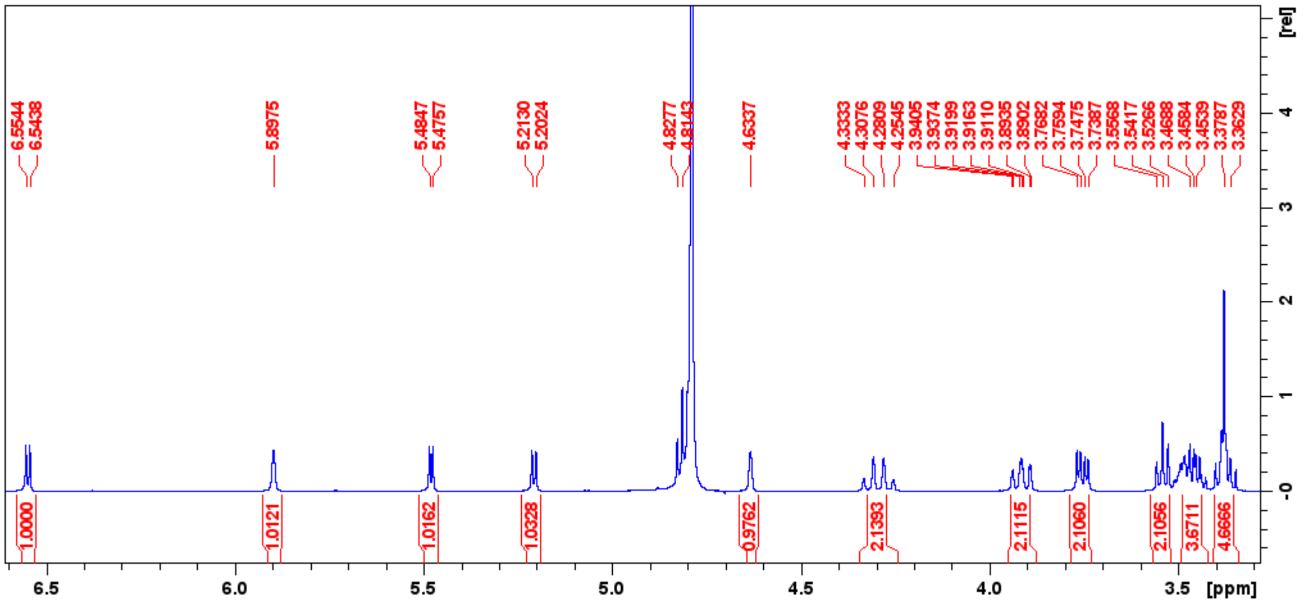

Figure S4. ^{1}H-NMR spectrum of melittoside (**2**) (^{1}H-NMR:600 MHz; D_2O)

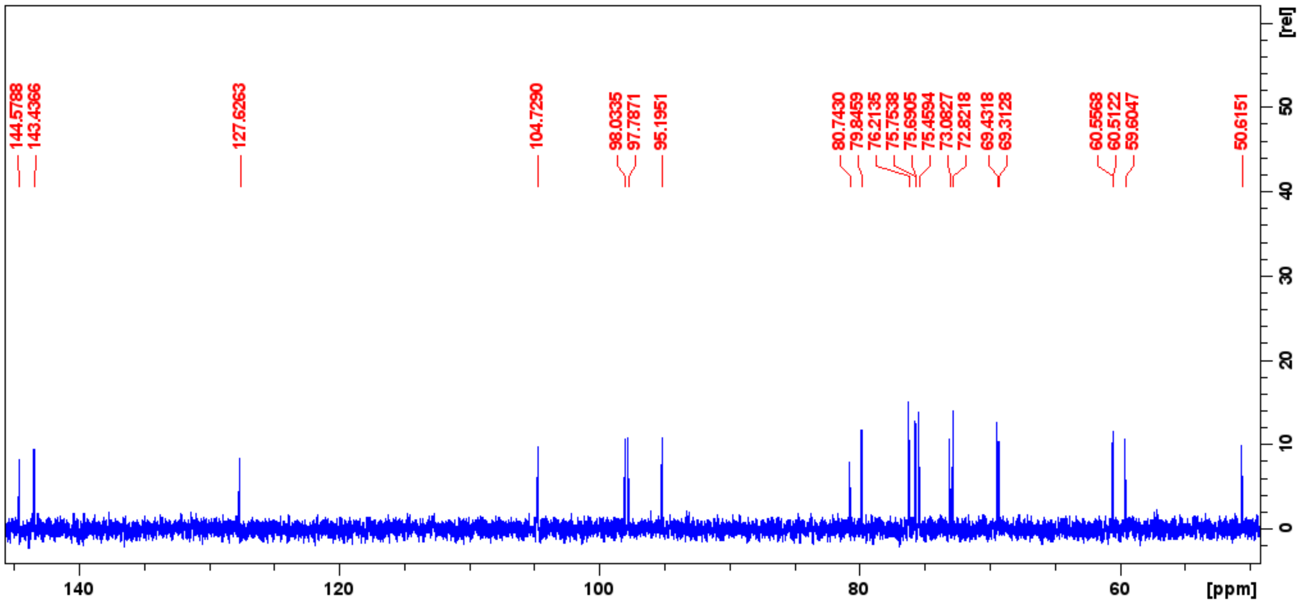

Figure S5. ^{13}C-NMR spectrum of melittoside (**2**) (^{13}C-NMR:150 MHz; D_2O)

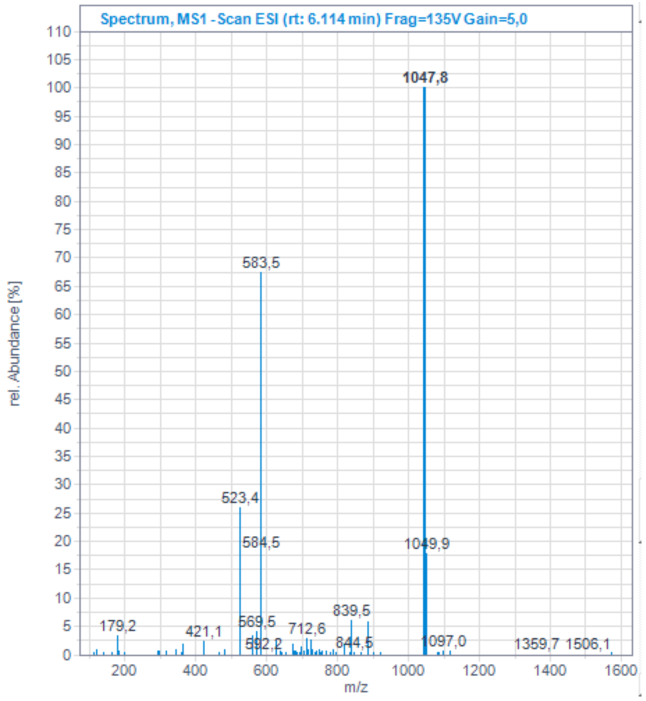

Figure S6. MS spectrum of melittoside (**2**) in negative mode. The major ions were 523 [M-H]$^-$, 583 [M+Hac-H]$^-$ and 1047 [2M-H]$^-$.

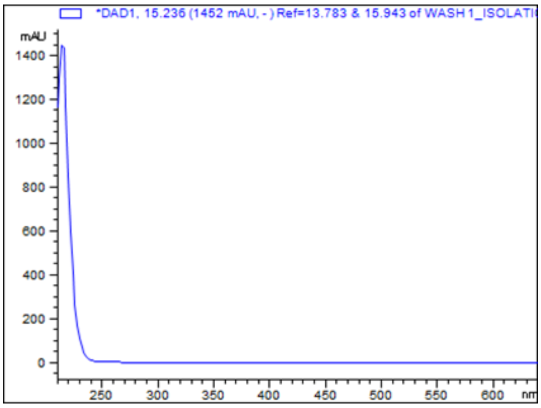

Figure S7. UV-vis spectrum of melittoside (**2**) showing a maximum absorbance at 210 nm.

Vanillic acid glucoside (3)

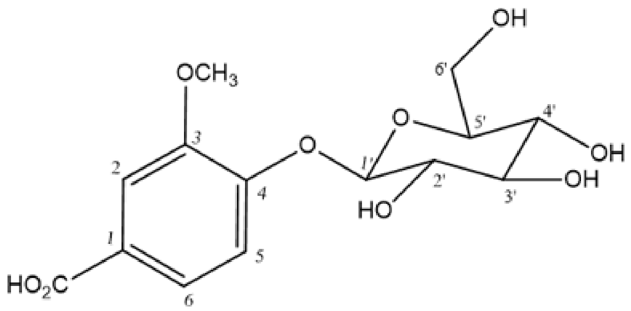

Table S3. ^{1}H-NMR (600 MHz) Data of vanillic acid glucoside (**3**) in D_2O.

Position	δ_H (ppm, *J* in Hz)
1	-
2	7.61 (d, *J*=1.9)
3	-
4	-
5	7.23 (d, *J*=8.5)
6	7.54 (dd, *J*=8.4, 1.9)
7	-
8	3.96 (s)
1'	5.24 (d, *J*=7.4)
2' 3' 5'	3.69-3.65 (m)**
4'	3.54-3.58 (m)
6a'	3.81 (d, *J*=5.5)
6b'	3.78 (d, *J*=5.4)

**assignments overlapped

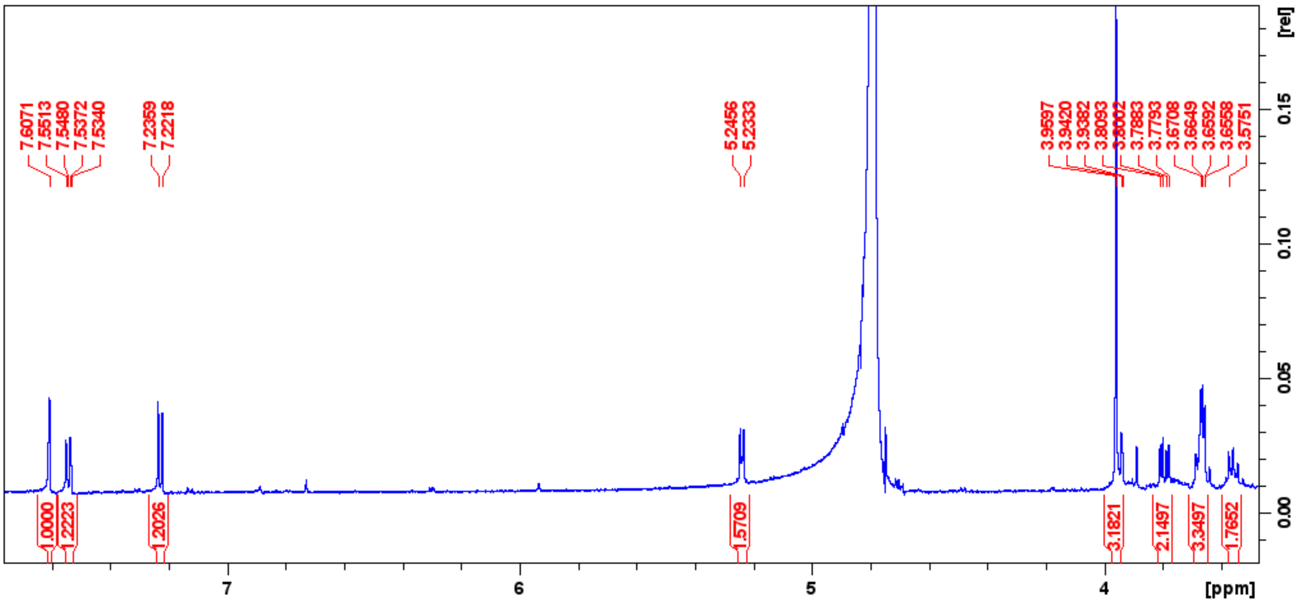

Figure S8. ^{1}H-NMR spectrum of vanillic acid glucoside (**3**) (^{1}H-NMR:600 MHz; D_2O)

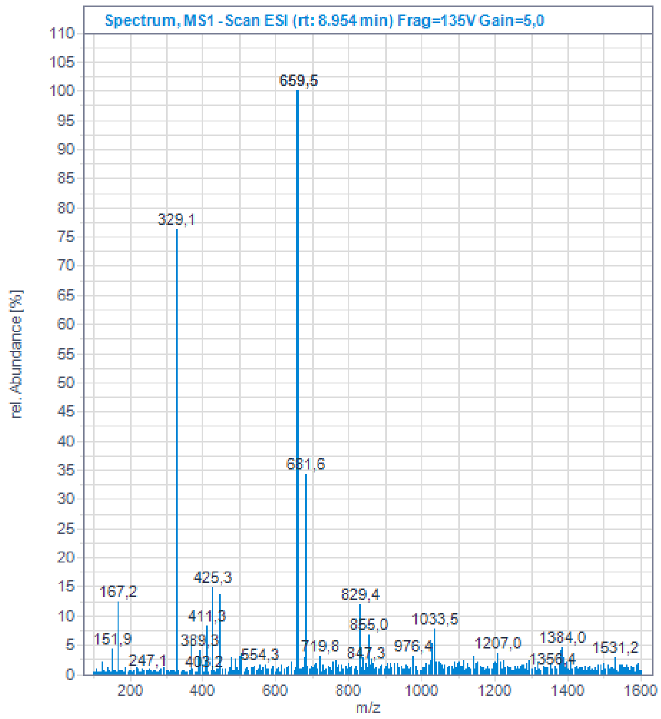

Figure S9. MS spectrum of vanillic acid glucoside (3) in negative mode. The major ions were 659 $[2M-H]^-$ and 329 [M-H]-.

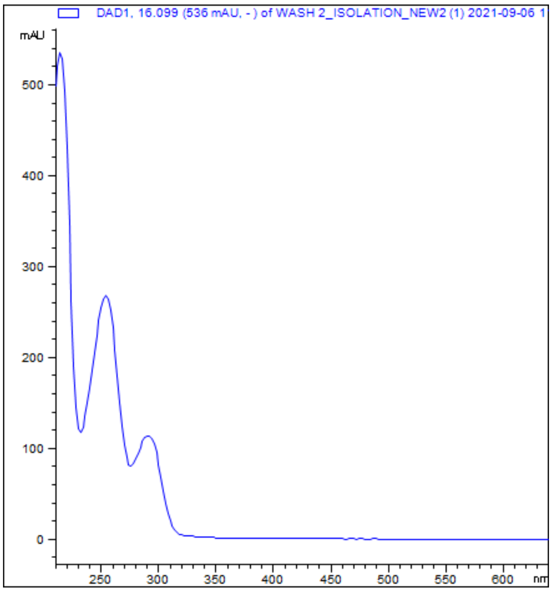

Figure S10. UV spectrum of vanillic acid glucoside (3). Maximum absorbance at 210, 254 and 290 nm.

Ajugoside (4)

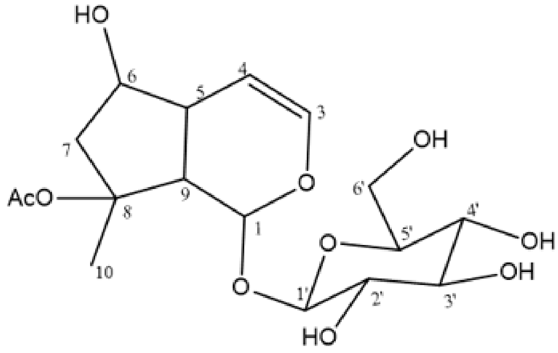

Table S4. ^{1}H-NMR (600 MHz) data of ajugoside (4) in D_2O

Position	δ_H (ppm, *J* in Hz)
1	5.93 (s)
3	6.30 (dd, *J*=6.3,2.1)
4	4.87 (d, *J*=6.4)
5	2.85 (d, *J*=8.3)
6	4.18 (d, *J*=4.3)
7a	2.19 (d, *J*=15.3)
7b	2.24 (d, *J*=15.5)
8	-
9	2.92 (d, *J*=8.4)
10	1.57 (s)
1'	*
2' 3' 4' 5' 6'	4.16-3.41 (m)**

*overlapped by the signal of the solvent **assignments overlapped

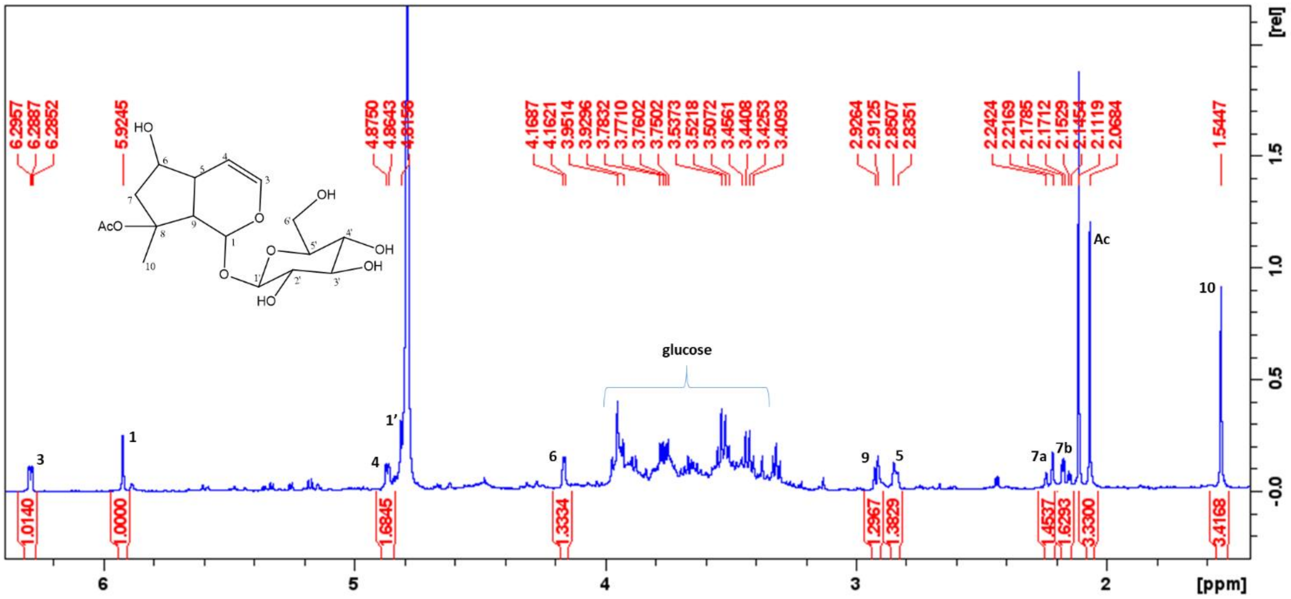

Figure S11. ^{1}H-NMR spectrum of ajugoside (4) (^{1}H-NMR:600 MHz; D_2O)

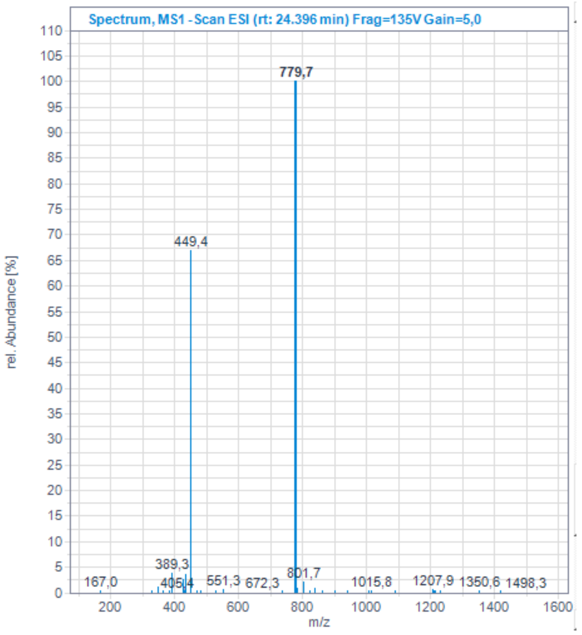

Figure S12. MS spectrum of ajugoside (4) in negative mode. The major ions were 449 [M+Hac-H]$^-$ and 779 [2M-H]-.

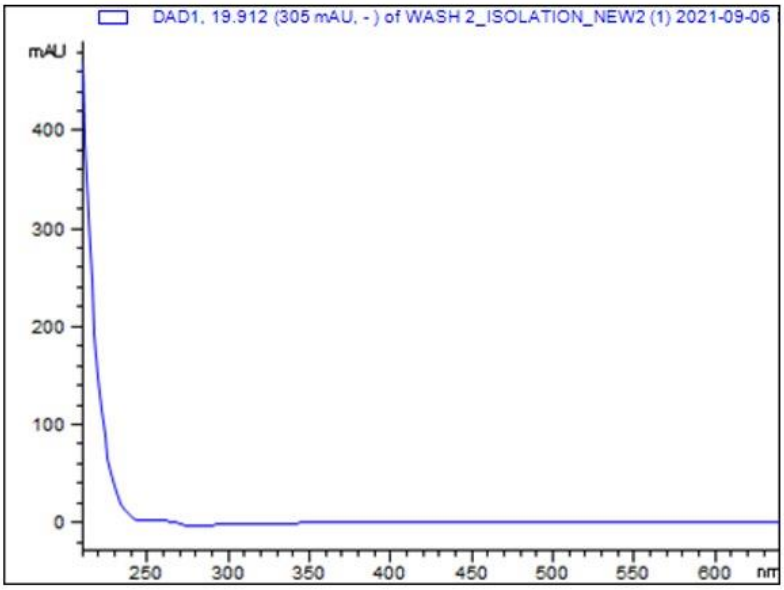

Figure S13. UV-vis spectrum of ajugoside (4). Maximum absorbance at 200 nm.

Salicylic acid glucoside (5)

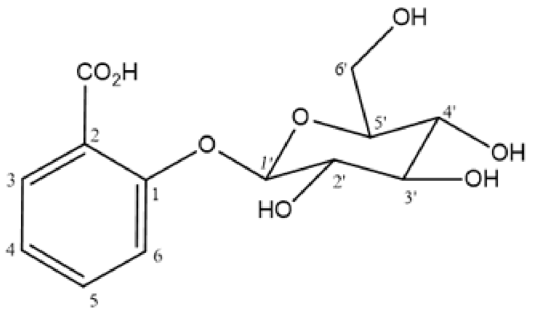

Table S5. ^{1}H-NMR data of salicylic acid glucoside (**5**) (D_2O).

Position	δ_H (ppm, *J* in Hz)
1	-
2	-
3	7.46-7.42 (m)**
4	7.18 (t, *J*=7.4)
5	7.46-7.42 (m)**
6	7.26 (d, *J*=8.3)
1'	5.10 (d, *J*=7.3)
2' 3' 4' 5'	3.66-3.52 (m)**
6a'	3.97 (dd, *J*=12.4, 2.1)
6b'	3.80 (dd, *J*=12.5, 5.7)

**assignments overlapped

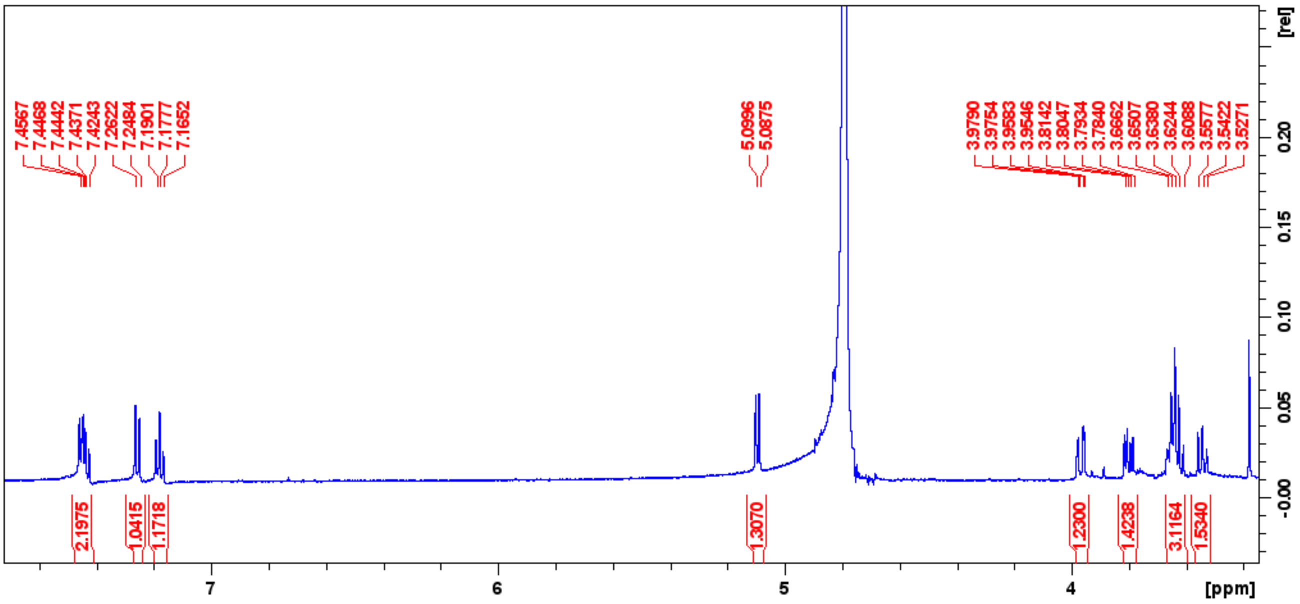

Figure S14. ^{1}H-NMR spectrum of salicylic acid glucoside (5) (^{1}H-NMR:600 MHz; D_2O)

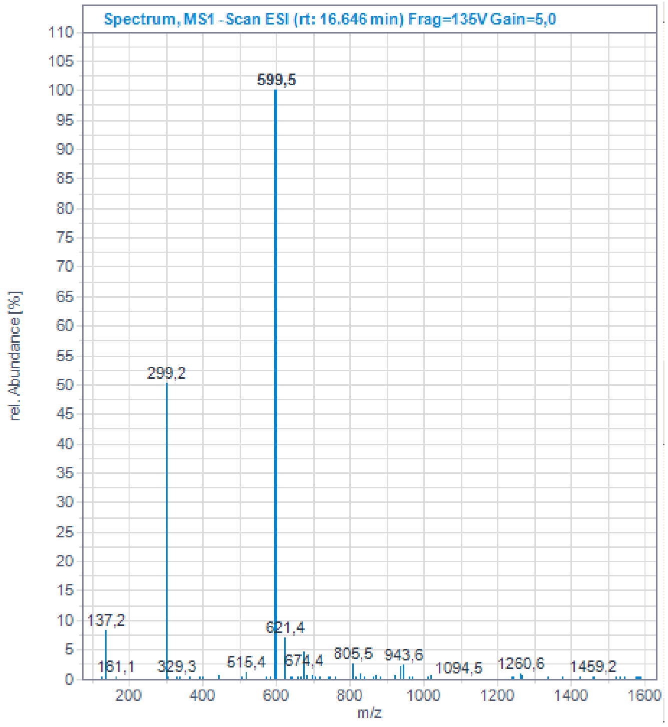

Figure S15. MS spectrum of salicylic acid glucoside (**5**) in negative mode. The major ions of 299 and 599 correspond to $[M-H]^-$ and $[2M-H]^-$.

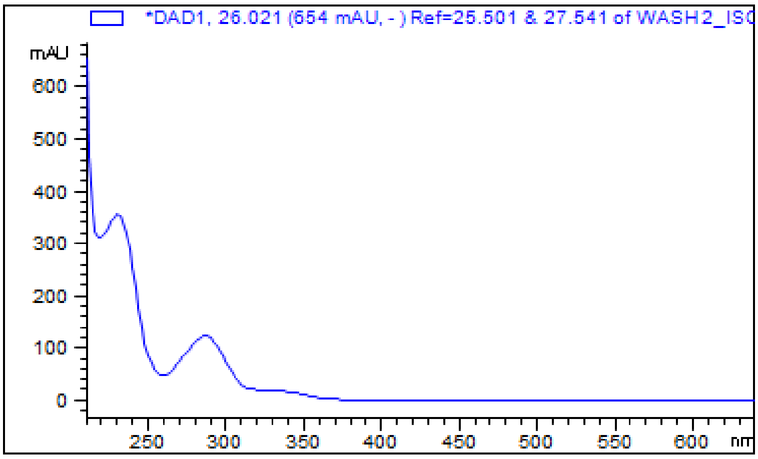

Figure S16. UV-vis spectrum of salicylic acid glucoside (**5**) having maximum absorbance at 230 and 286 nm.

7-O-acetyl-8-epi-loganic acid (6)

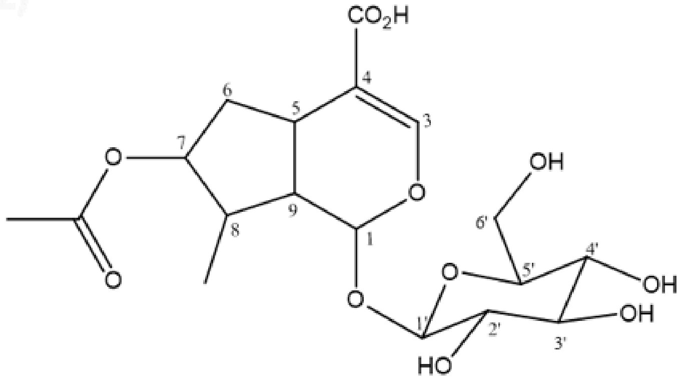

Table S6. ^{1}H-NMR data of 7-O-acetyl-8-epi-loganic acid (6) (D_2O)

**Position**	**δ_H (ppm, *J* in Hz)**
1	5.60 (d, *J*=2.7)
3	7.32 (s)
4	-
5	3.09 (m)
6a	1.98 (dt, *J*=14.4, 11.8)
6b	2.23 (m)
7	*
8	2.46 (m)
9	2.75 (m)
10	1.03 (d, *J*=7.5)
1'	*
2'	3.30 (dd, *J*=9.3, 8.2)
3'	3.55-3.41 (m)**
4'	3.55-3.41 (m)**
5'	3.55-3.41 (m)**
6a'	3.76 (dd, *J*=12.4, 6.0)
6b'	3.96 (dd, *J*=12.3, 2.1)
COCH3	2.1 (s)

*overlapped by the signal of the solvent **assignments overlapped

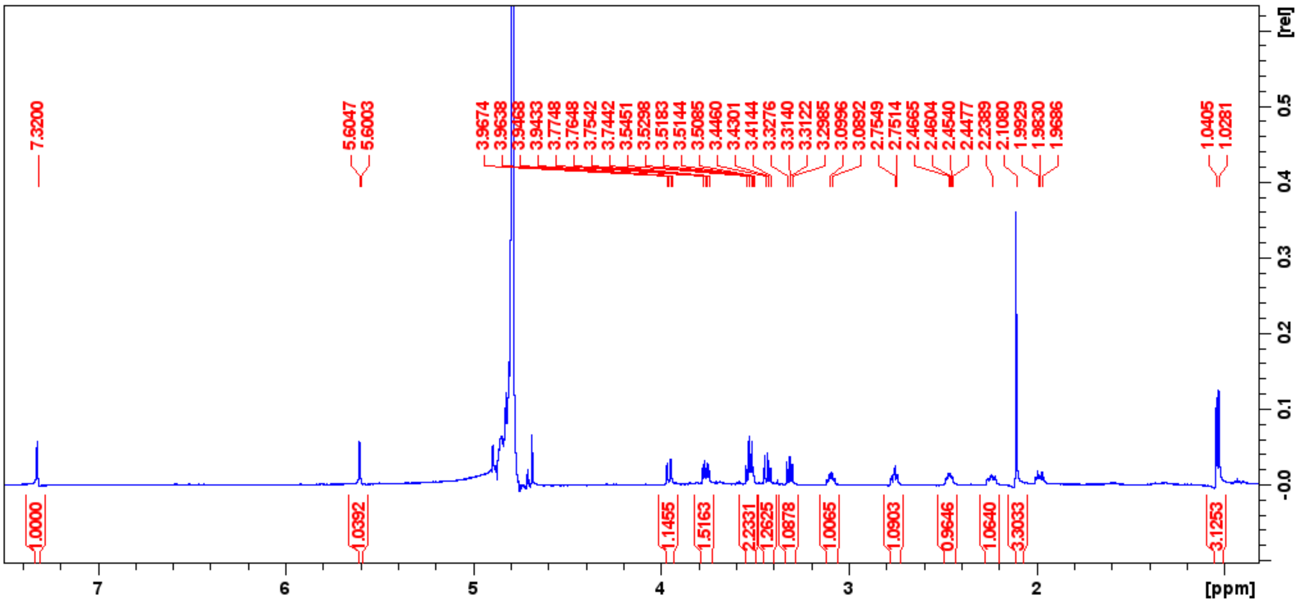

Figure S17 ^{1}H-NMR spectrum of 7-O-acetyl-8-epi-loganic acid (**6**) (^{1}H-NMR:600 MHz; D_2O)

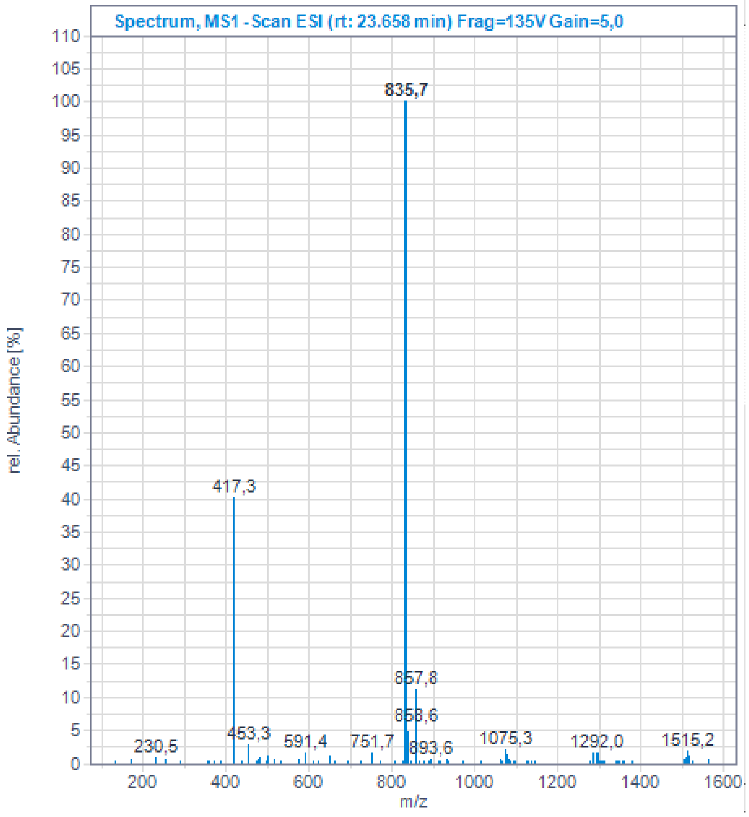

Figure S18. MS spectrum of 7-O-acetyl-8-epi-loganic acid (**6**) in negative mode. The major ions were 417 $[M-H]^-$ and 835 $[2M-H]^-$.

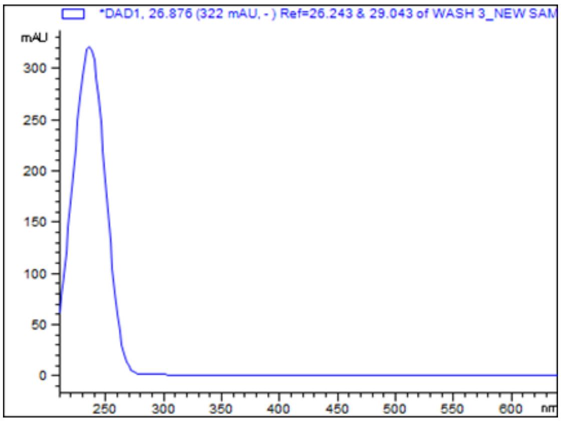

Figure S19. UV spectrum of 7-O-acetyl-8-epi-loganic acid (**6**) with maximum absorbance at 236 nm.

Chlorogenic acid (7)

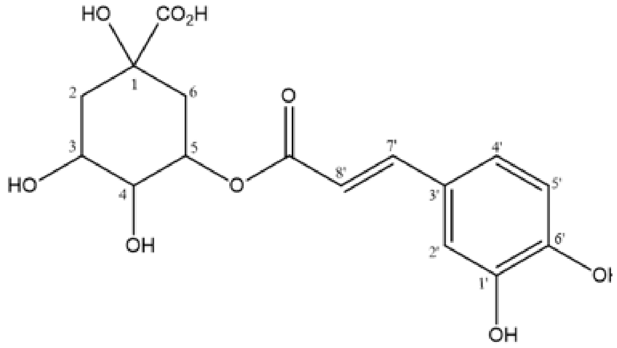

Table S7. ^{1}H-NMR data of chlorogenic acid (7) (CD_3OD)

Position	δ_H (ppm, *J* in Hz)
1'	-
2'	7.04 (d, *J*=1.8)
3'	-
4'	-
5'	6.77 (d, *J*=8.2)
6'	6.94 (dd, *J*=8.1, 1.6)
7'	7.56 (d, *J*=15.9)
8'	6.28 (d, *J*=15.9)
9'	-
1	-
2	2.22-2.01 (m)**
3	4.16 (brs)
4	3.71 (dd, *J*=9.2, 2.2)
5	5.35-5.31 (m)
6	2.23-2.03 (m)**

**assignments overlapped

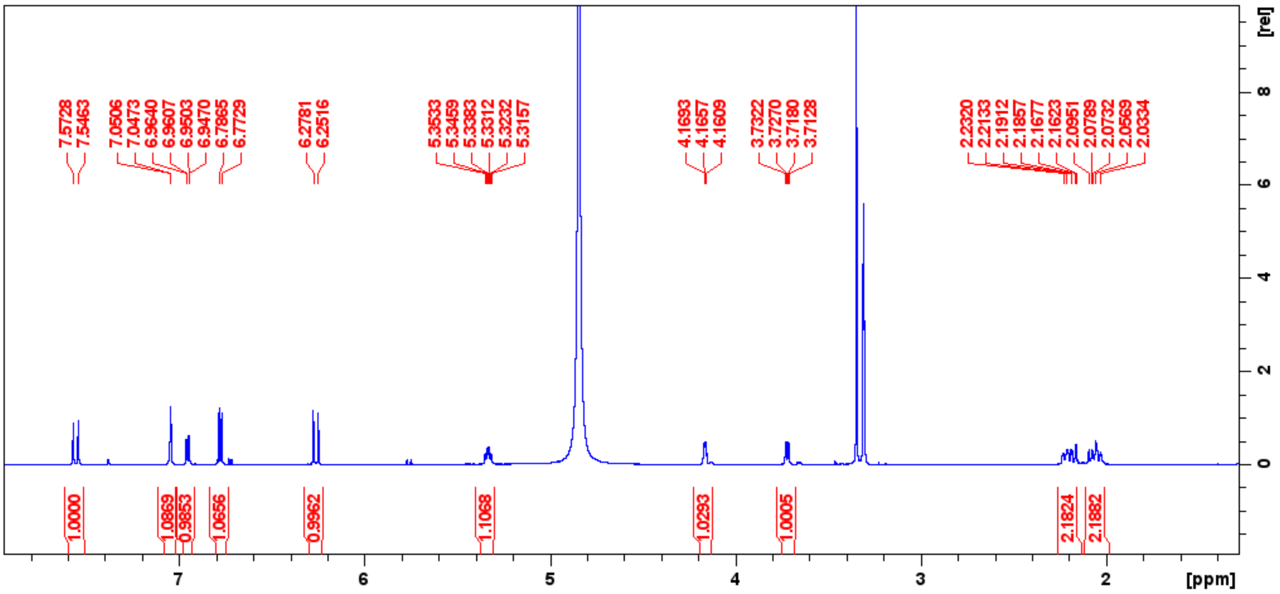

Figure S20. ^{1}H-NMR spectrum of chlorogenic acid (7) (^{1}H-NMR:600 MHz; CD_3OD)

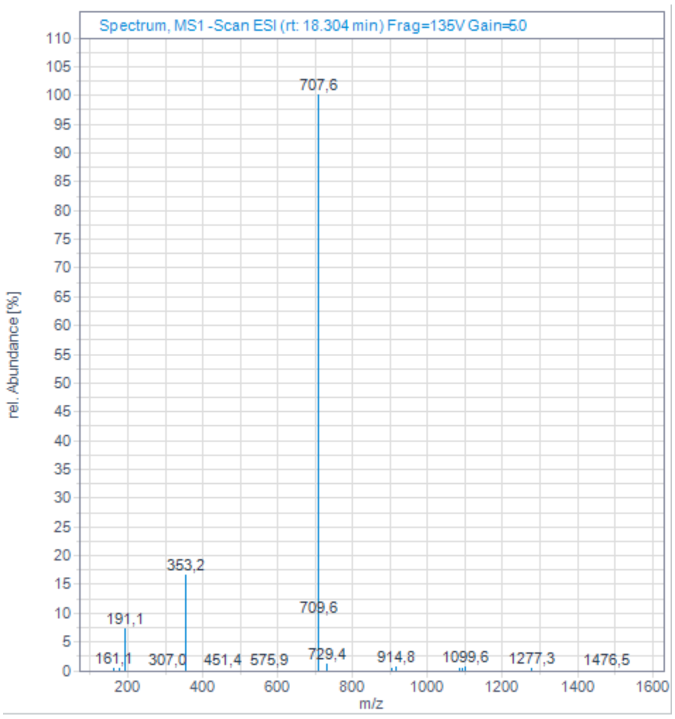

Figure S21. MS spectrum of chlorogenic acid (7) in negative mode. The major ions of 707 and 353 correspond to [2M-H]- and [M-H]-, whereas the ion of m/z=191 is a fragment corresponding to quinic acid.

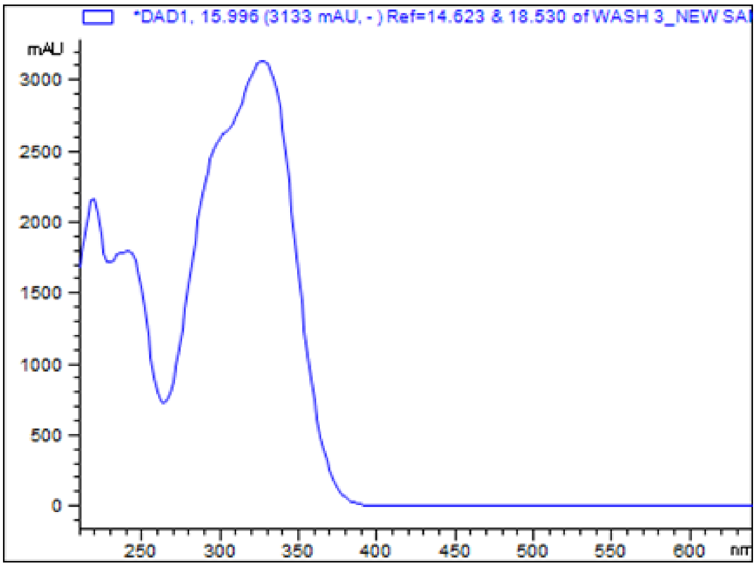

Figure S22. UV-vis spectrum of chlorogenic acid (**7**) showing maximum absorbance at 330 nm.

Verbascoside (8)

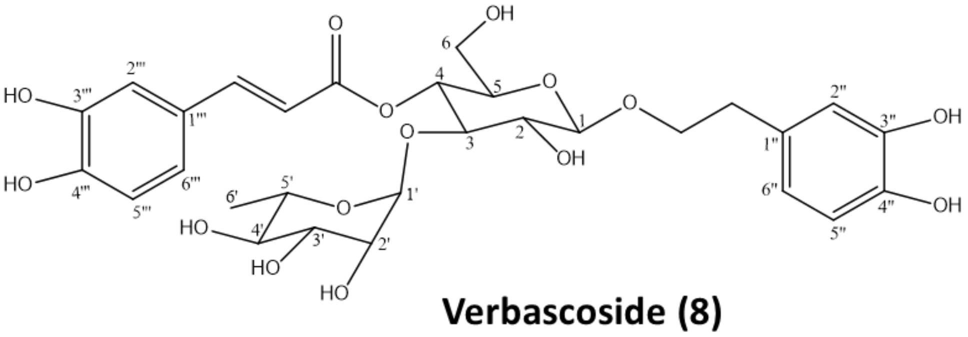

Verbascoside (8)

Table S8. ^{1}H-NMR, ^{13}C-NMR and APT-NMR data of verbascoside (8) (D_2O)

**Position**	**δ_H (ppm, *J* in Hz)**	APT	**δ_C (ppm)**
1'''		C	126.16
2'''	7.12 (d, *J*=1.9)	CH	115.18
3'''		C	147.73
4'''		C	147.52
5'''	6.86 (d, *J*=8.2)	CH	116.23
6'''	7.06 (dd, *J*=8.3, 2)	CH	123.20
7'''	7.61 (d, *J*=15.9)	CH	147.52
8'''	6.31 (d, *J*=15.9)	CH	113.67
1''		C	131.46
2''	6.79-6.78 (m)	CH	116.75
3''		C	143.88
4''		C	142.31
5''	6.79-6.78 (m)	CH	116.29
6''	6.68 (dd, *J*=8.1, 1.8)	CH	121.20
7''	2.75 (t, *J*=6.7)	CH_2	34.43
8''	4.03-3.98 (m), 3.81-3.77 (m)	CH_2	71.08
1	4.41 (d, *J*=8.1)	CH	102.02
2	3.43-3.37 (m)	CH	73.92
3	3.76-3.75 (m)	CH	80.76
4	4.88 (t, *J*=9.6)	CH	68.76
5	3.58-3.53 (m)	CH	73.64
6	3.62 (dd, *J*=12.3, 2.1), 3.49-3.46 (m)	CH_2	60.10
1'	5.03 (d, *J*=1.5)	CH	101.57
2'	3.92-3.91 (m)	CH	70.25
3'	3.58-3.53 (m)	CH	69.82
4'	3.25 (t, *J*=9.6)	CH	71.72
5'	3.43-3.37 (m)	CH	69.43
6'	0.96 (d, *J*=6.2)	CH_2	17.08

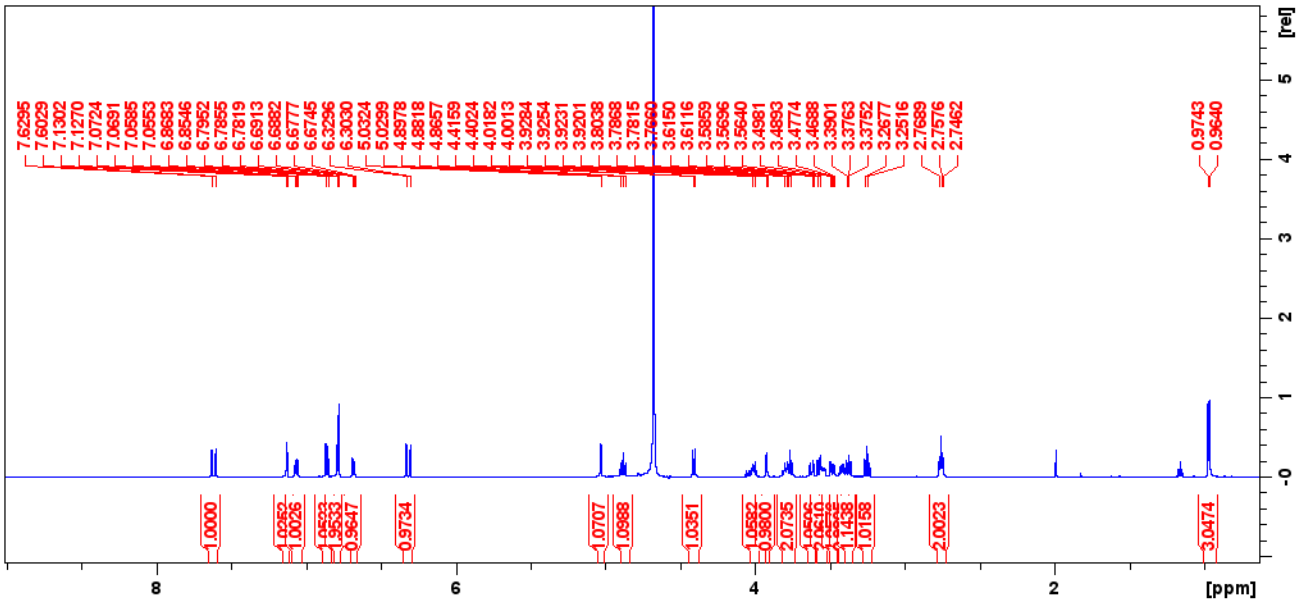

Figure S23. ^{1}H-NMR spectrum of verbascoside (8) (^{1}H-NMR:600 MHz; D_2O)

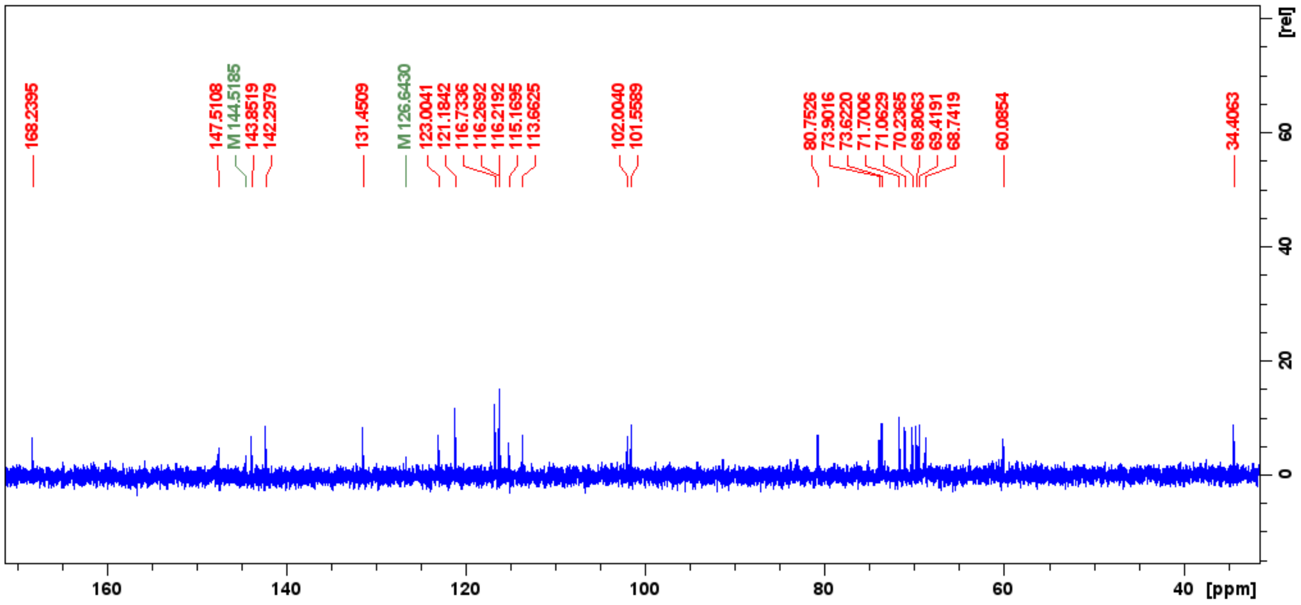

Figure S24. ^{13}C-NMR spectrum of verbascoside (8) (^{13}C-NMR:150 MHz; D_2O)

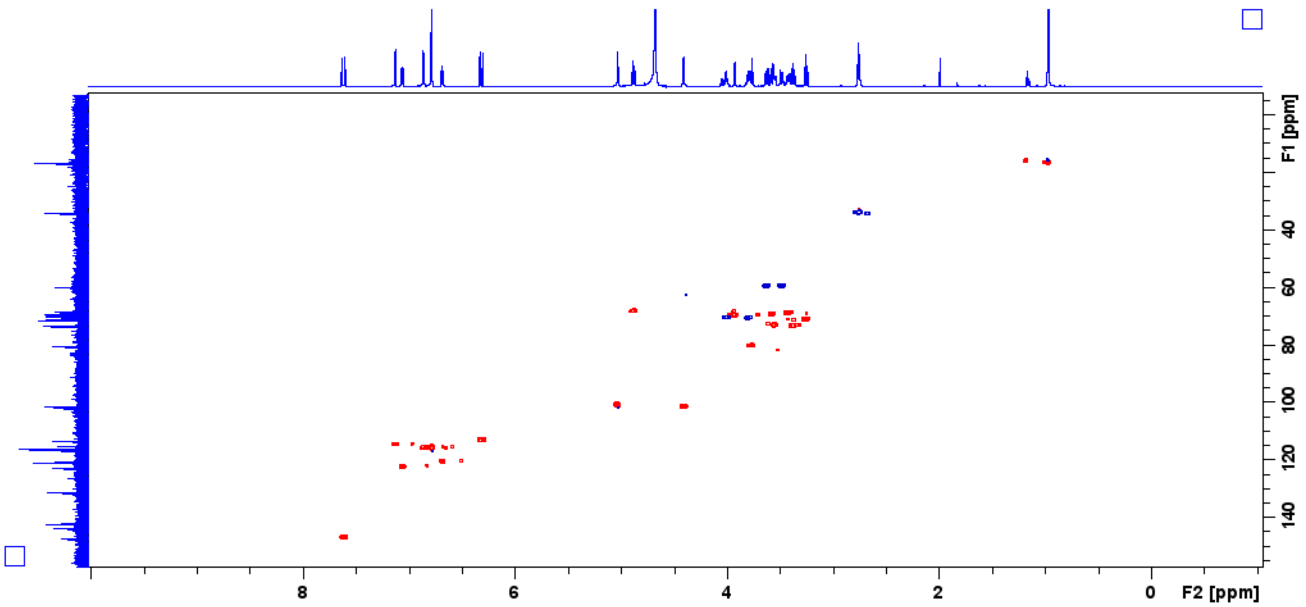

Figure S25. HSQC-NMR spectrum of verbascoside (8) (D_2O)

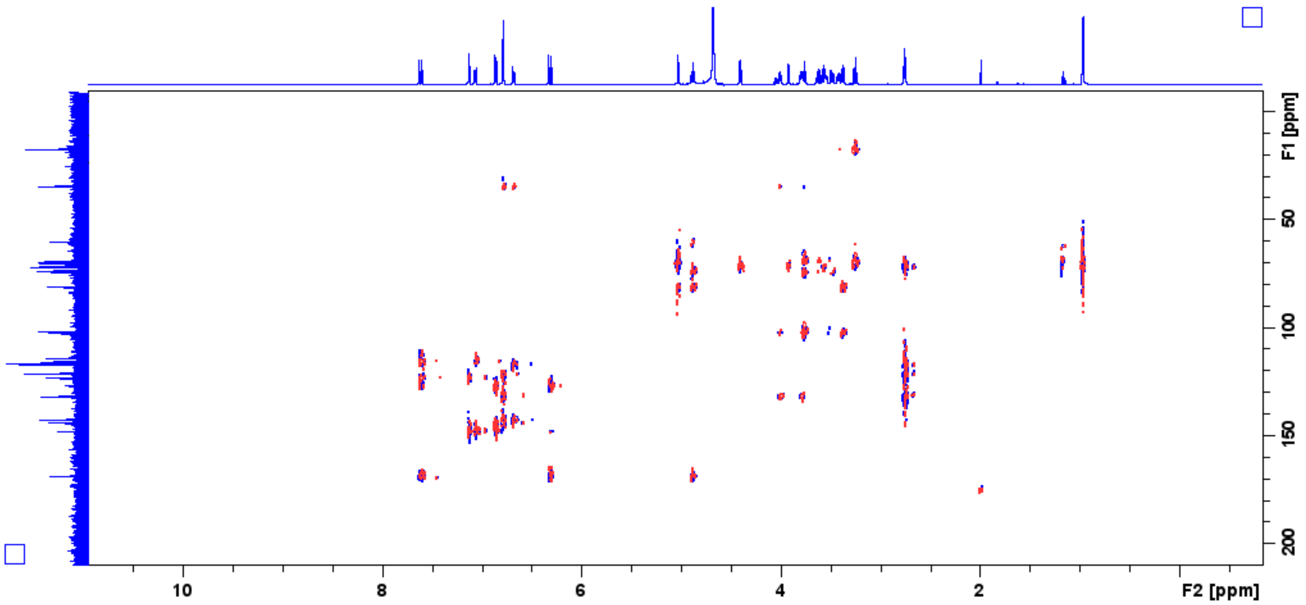

Figure S26. HMBC-NMR spectrum of verbascoside (8) (D_2O)

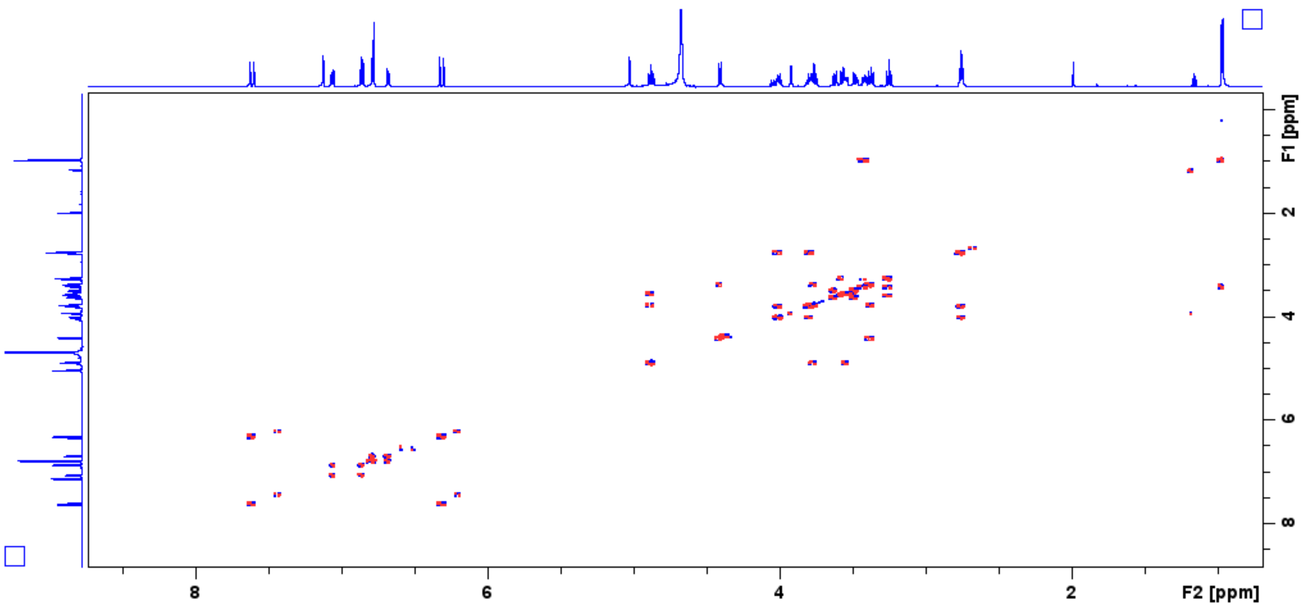

Figure S27. COSY-NMR spectrum of verbascoside (8) (D_2O)

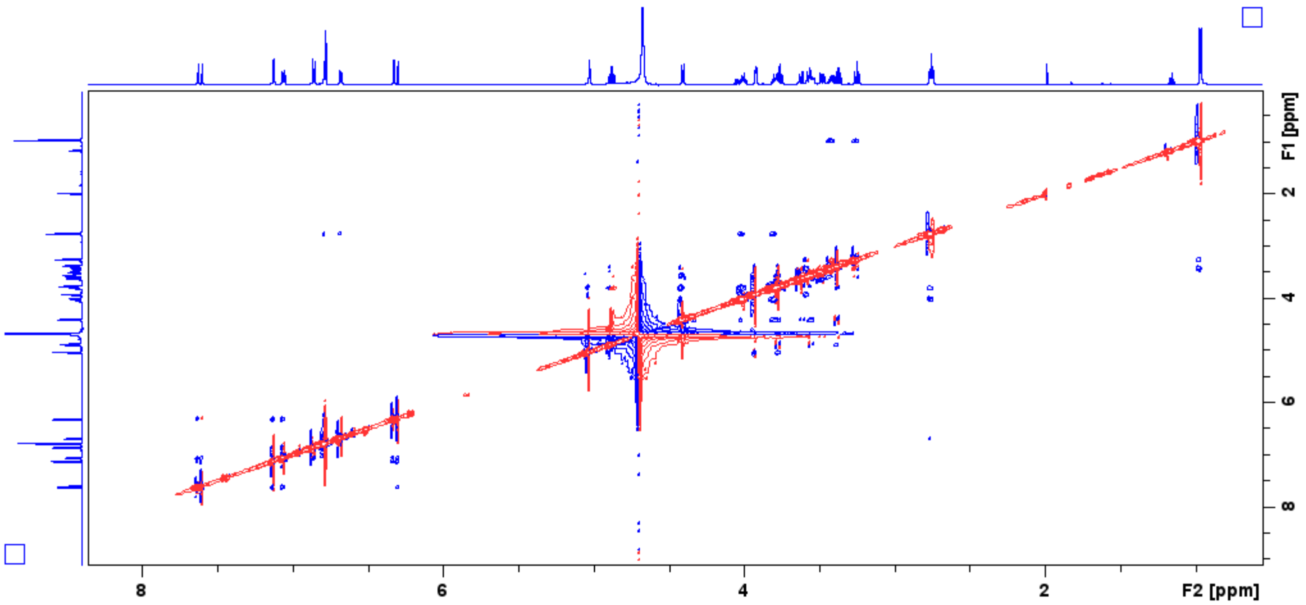

Figure S28. ROESY- NMR spectrum of verbascoside (8) (D_2O)

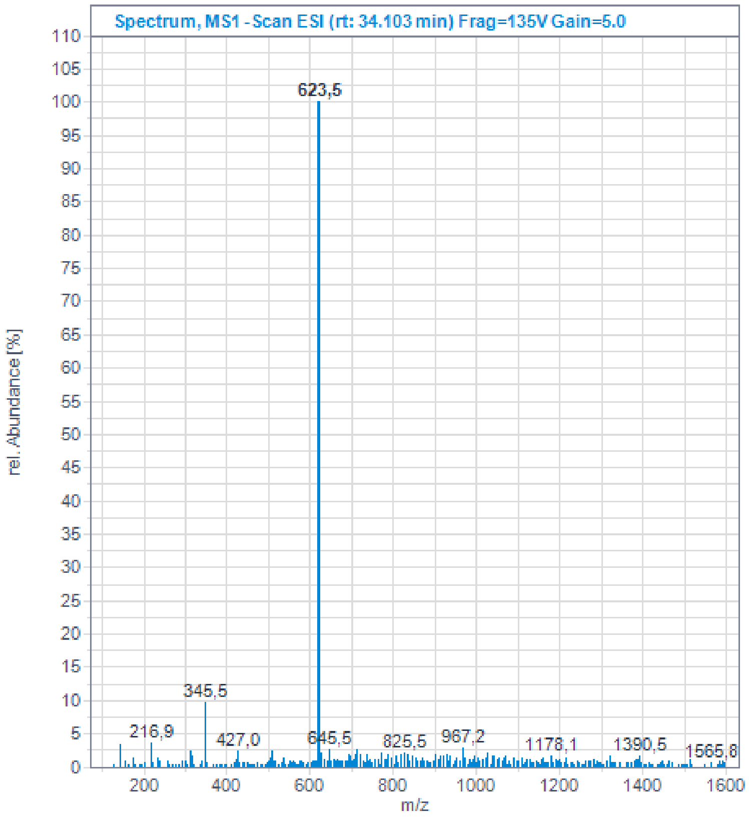

Figure S29. MS spectrum of verbascoside (8) in negative mode with the sole ion of 623 [M-H]$^{-}$.

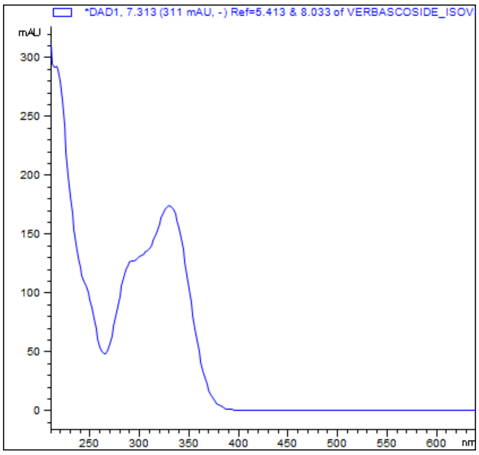

Figure S30. UV spectrum of verbascoside (8) showing maximum absorbance at 210 and 330 nm.

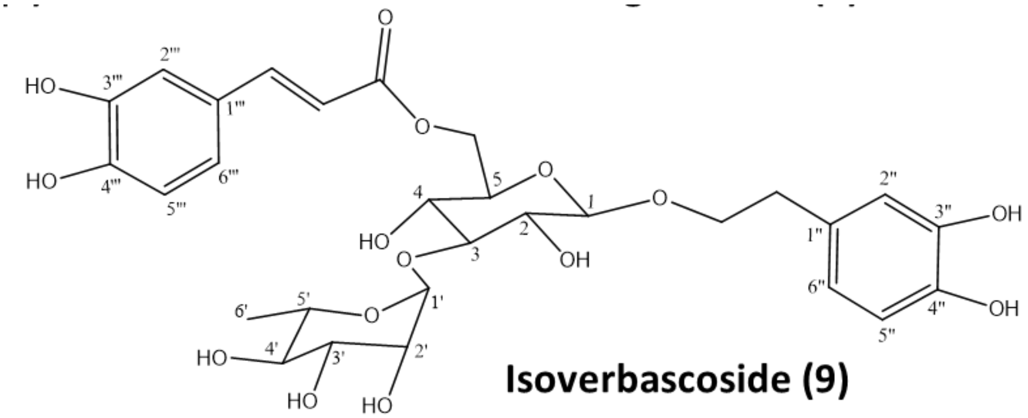

Isoverbascoside (9)

Table S9. ^{1}H-NMR, ^{13}C-NMR and APT-NMR data of isoverbascoside (9) (D_2O)

Position	δ_H (ppm, *J* in Hz)	APT	δ_C (ppm)
1'''		C	126.68
2'''	7.01 (s)	CH	115.18
3'''		C	147.15
4'''		C	144.16
5'''	6.83 (s)	CH	116.01
6'''	6.83 (s)	CH	122.69
7'''	7.46 (d, *J*=16)	CH	146.29
8'''	6.23 (d, *J*=16)	CH	113.95
1''		C	130.77
2''	6.72 (s)	CH	116.58
3''		C	143.73
4''		C	142.20
5''	6.68 (d, *J*=7.9)	CH	116.01
6''	6.56 (d, *J*=7.7)	CH	121.01
7''	2.74 (t, *J*=7.3)	CH_2	34.82
8''	3.91-3.87 (m), 3.83-3.76 (m)	CH_2	71.45
1	4.50-4.44 (m)	CH	102.2
2	3.42 (t, *J*=8.5)	CH	73.62
3	3.62 (t, *J*=9)	CH	82.32
4	3.53-3.47 (m)	CH	68.69
5	3.69 (t, *J*=9)	CH	73.38
6	4.50-4.44 (m), 4.40-4.37 (m)	CH_2	63.40
1'	5.16 (s)	CH	101.09
2'	4.08 (s)	CH	70.32
3'	3.83-3.76 (m)	CH	70.17
4'	3.53-3.47 (m)	CH	71.96
5'	4.06-4.02 (m)	CH	68.85
6'	1.29 (d, *J*=6.2)	CH_2	16.49

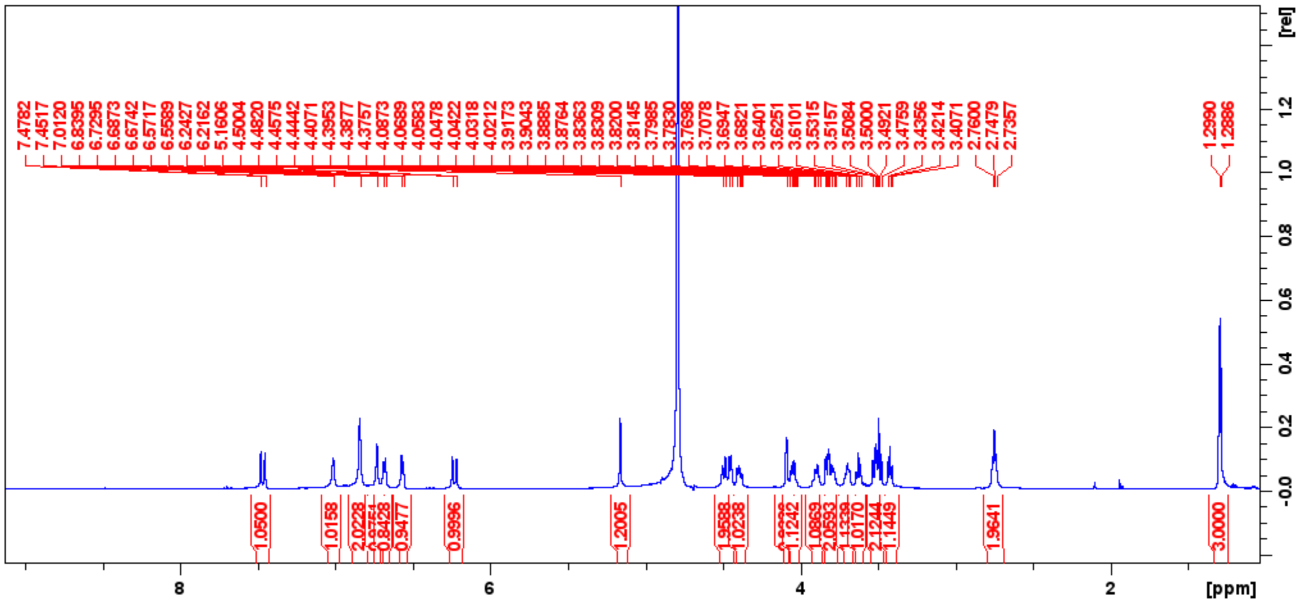

Figure S31. ^{1}H-NMR spectrum of isoverbascoside (9) (^{1}H-NMR:600 MHz; D_2O)

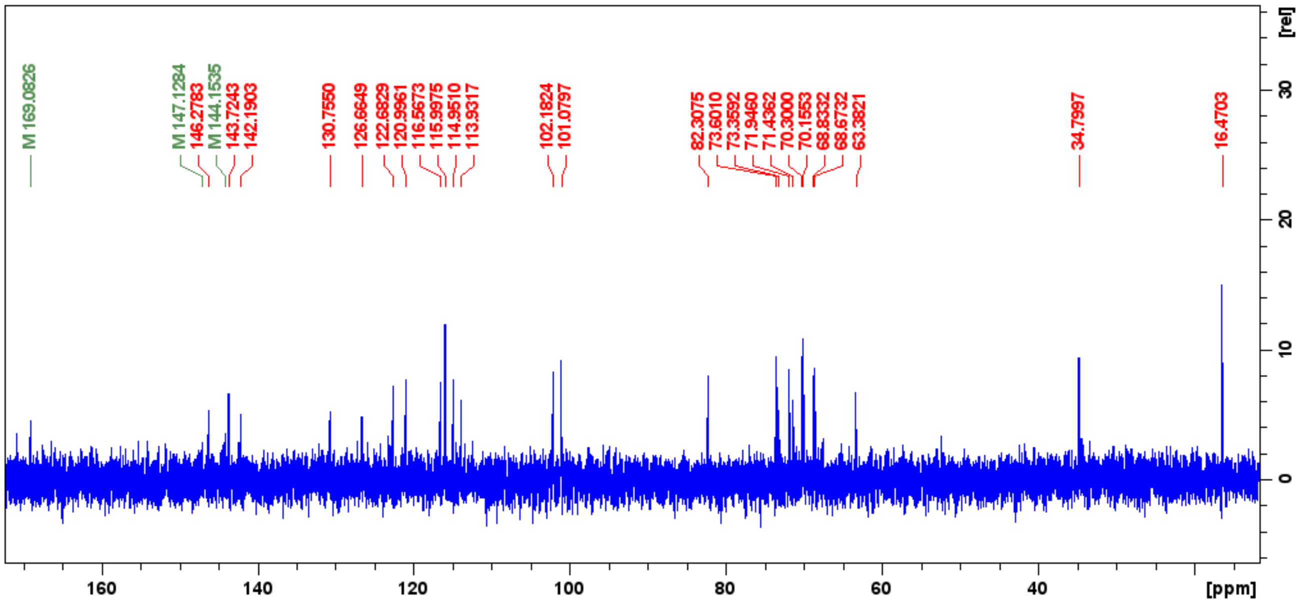

Figure S32. ^{13}C-NMR spectrum of isoverbascoside (9) (^{13}C-NMR:150 MHz; D_2O)

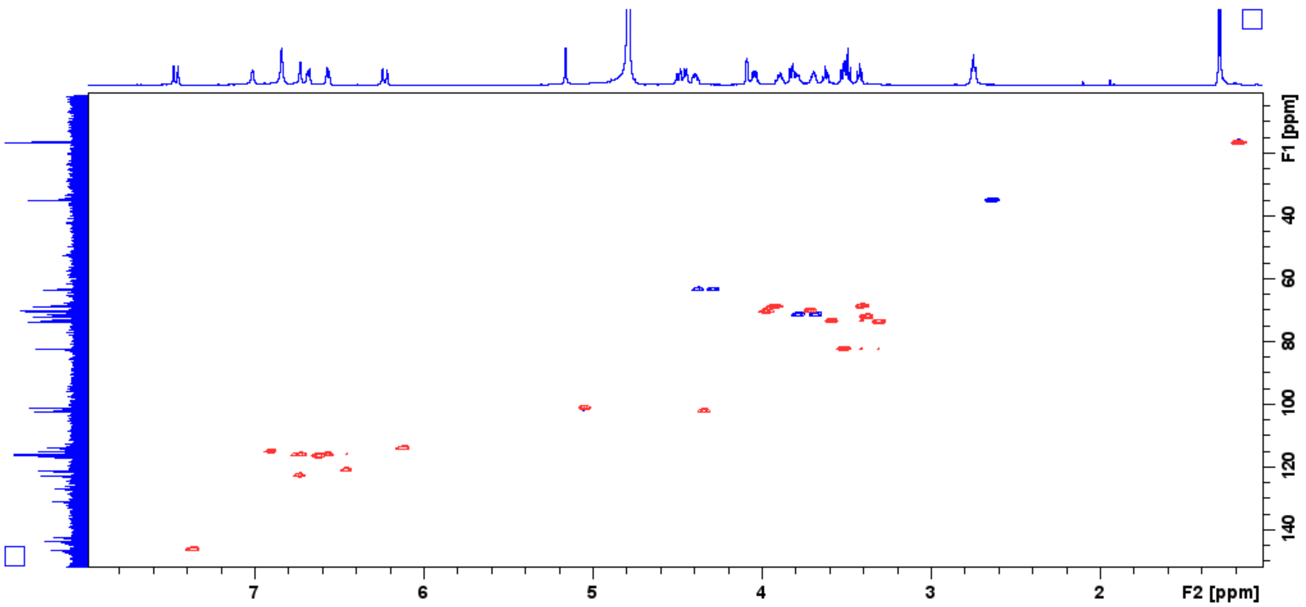

Figure S33. HSQC-NMR spectrum of isoverbascoside (9) (D_2O)

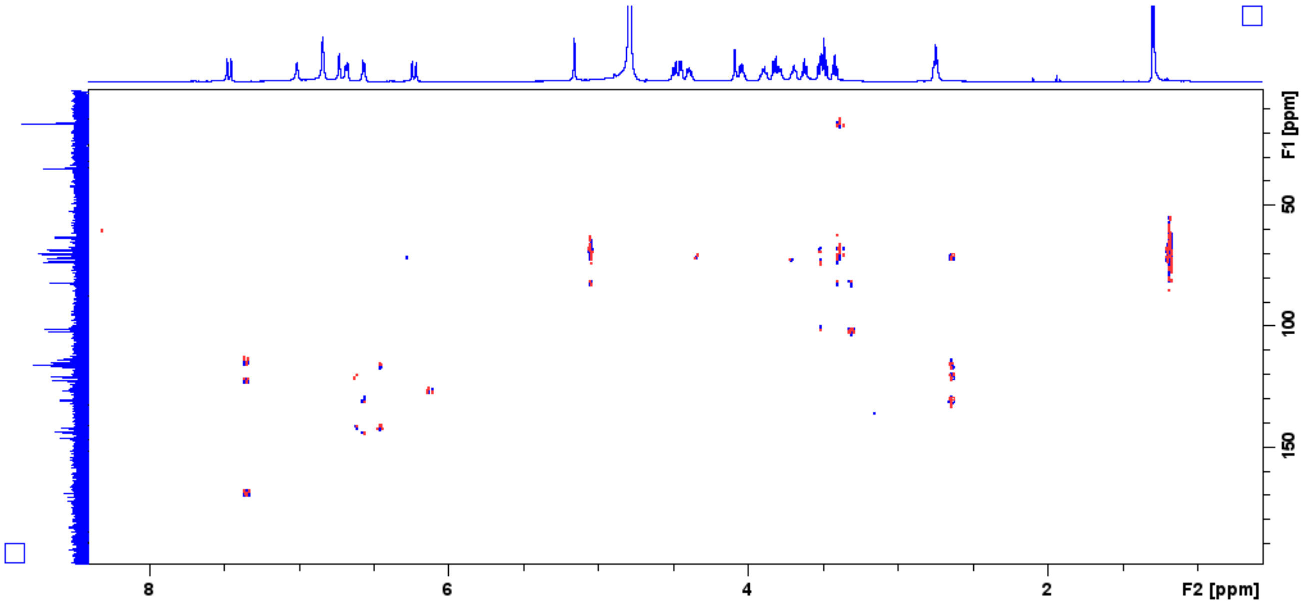

Figure S34. HMBC-NMR spectrum of isoverbascoside (9) (D_2O)

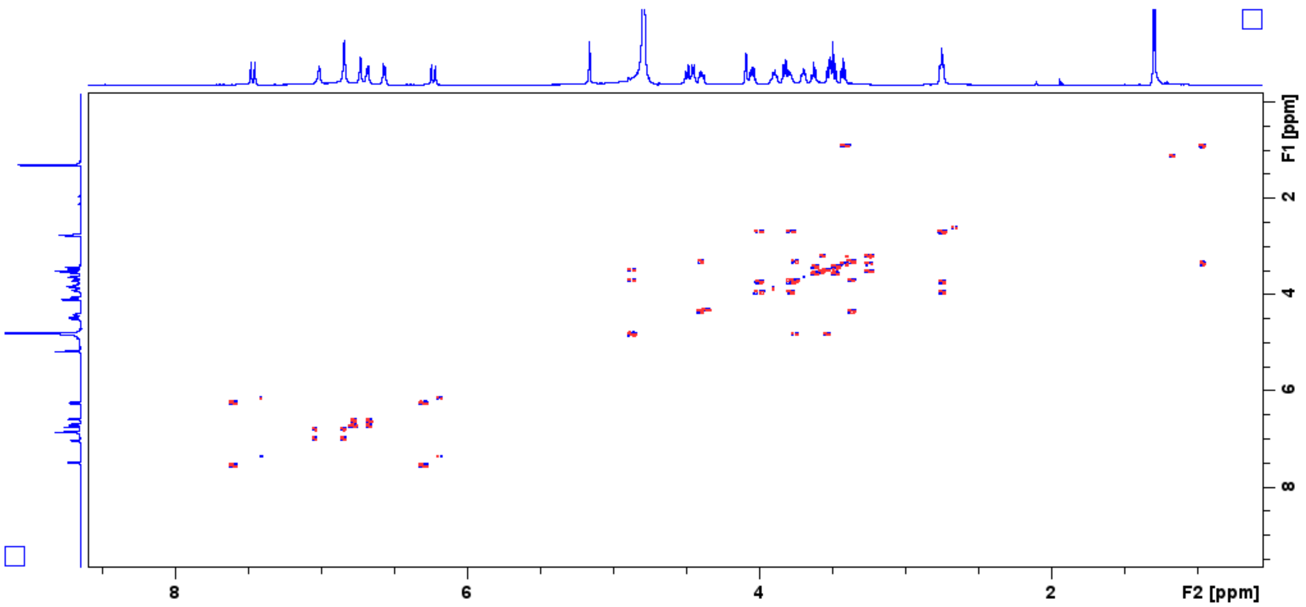

Figure S35. COSY-NMR spectrum of isoverbascoside (9) (D_2O)

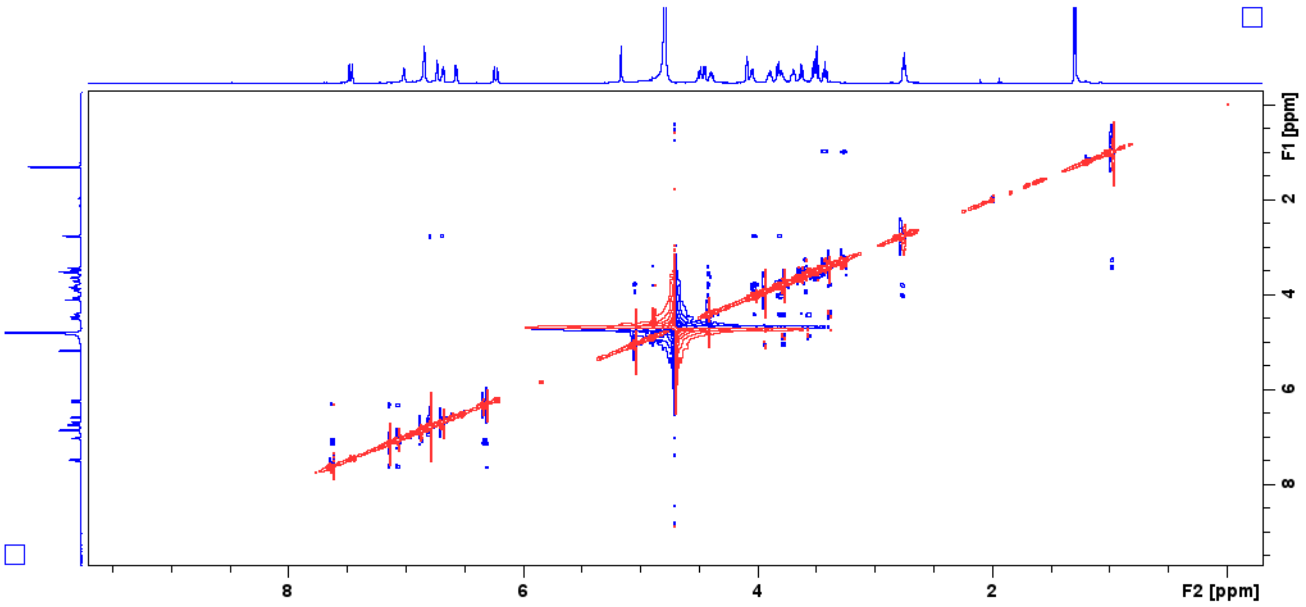

Figure S36. ROESY- NMR spectrum of isoverbascoside (9) (D_2O)

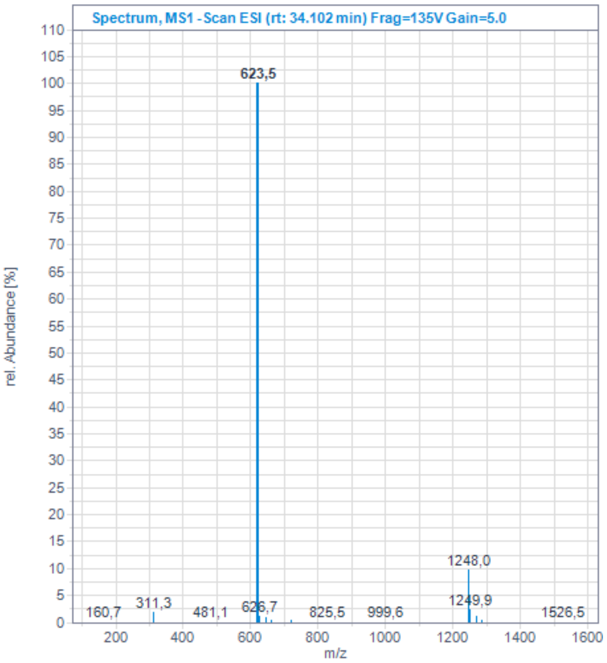

Figure S37. MS spectrum of isoverbascoside (9) in negative mode with the main ion of of 623 [M-H]$^{-}$.

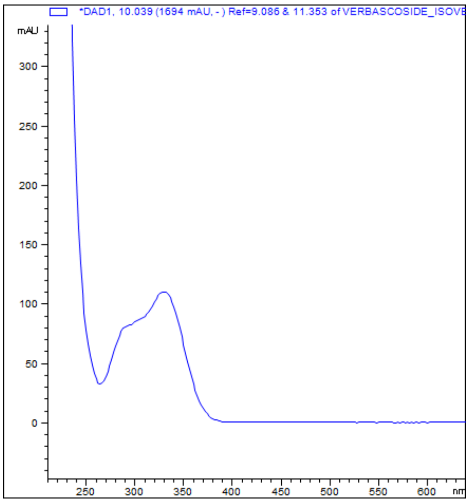

Figure S38. UV-vis spectrum of isoverbascoside (9) showing absorbance maxima at 210 and 330 nm.

Part B. Other Data

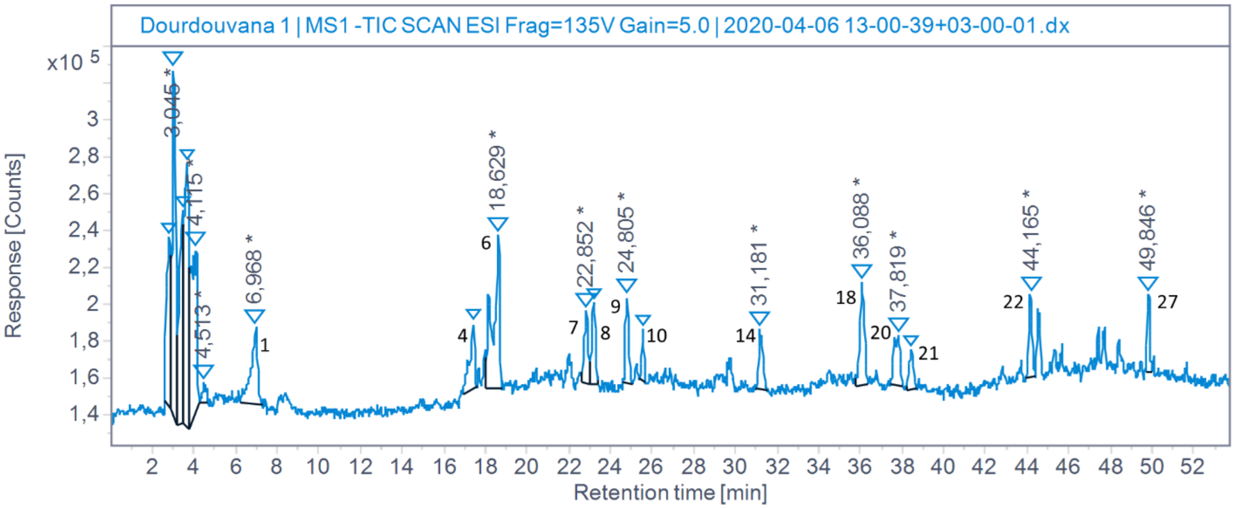

Figure S39. Total ion chromatogram (TIC) in negative mode of *Sideritis clandestina* subsp. *peloponnesiaca* (SCP) aqueous extract.
